# 1,3-Dipolar Cycloaddition Reactions of Azomethine Ylides with Carbonyl Dipolarophiles Yielding Oxazolidine Derivatives

**DOI:** 10.3390/molecules21080935

**Published:** 2016-07-23

**Authors:** Adam G. Meyer, John H. Ryan

**Affiliations:** CSIRO Manufacturing, Bag 10, Clayton South MDC, Victoria 3169, Australia; adam.meyer@csiro.au

**Keywords:** dipolar, cycloaddition, azomethine, ylide, carbonyl, aldehyde, ketone, anhydride, oxazolidine, spiro

## Abstract

We provide a comprehensive account of the 1,3-dipolar cycloaddition reactions of azomethine ylides with carbonyl dipolarophiles. Many different azomethine ylides have been studied, including stabilized and non-stabilized ylides. Of the carbonyl dipolarophiles, aldehydes including formaldehyde are the most studied, although there are now examples of cycloadditions with ketones, ketenes and carboxyl systems, in particular isatoic anhydrides and phthalic anhydrides. Intramolecular cycloadditions with esters can also occur under certain circumstances. The oxazolidine cycloadducts undergo a range of reactions triggered by the ring-opening of the oxazolidine ring system.

## 1. Introduction

The versatile and convergent nature of the 1,3-dipolar cycloaddition reaction has led to its development as a powerful method for the synthesis of five-membered heterocycles [1,2,3,4,5,6,7]. The reaction involves the addition of 1,3-dipoles, such as azides, nitrones, carbonyl ylides, nitrile oxides, nitrile imines and azomethine ylides to unsaturated double or triple bonds (1,2-dipolarophiles) [8,9,10,11,12,13,14,15]. Azomethine ylides **1**, which contain four electrons distributed over the π orbitals of a C-N-C group, are examples of the bent allyl anion-type of 1,3-dipole [16,17,18]. Azomethine ylides can be classified as non-stabilized (where R^1^–R^5^ = H or alkyl) or stabilized either by electron-withdrawing/electron-donating groups at the appropriate termini of the ylide or by *N*-metalation [18]. Azomethine ylides are mostly generated in situ due to their high reactivity and/or transient existence; however, in some cases, stabilized ylides have been isolated [19,20,21]. The most frequent type of 1,3-dipolar cycloaddition reaction of azomethine ylides is that with alkenyl or alkynyl dipolarophiles substituted with electron-withdrawing groups, providing access to pyrrolidine-containing molecules of biological [18,22,23,24,25,26] or materials science interest [27,28,29,30,31]. The reactions with multiple bonded heteroatom systems such as carbonyl, thiocarbonyl, isothiocyanato, imino, isocyanato, nitrile, nitroso, and azo derivatives are also known, but less well studied [7,16,32,33,34,35,36,37,38]. Less recognized is the ability of azomethine ylides to react with aromatic dipolarophiles when the aromatic system is embedded within a polycyclic aromatic hydrocarbon, tethered with the azomethine ylide (an intramolecular process) or substituted with highly electron withdrawing nitro groups [39].

**Scheme 1:**
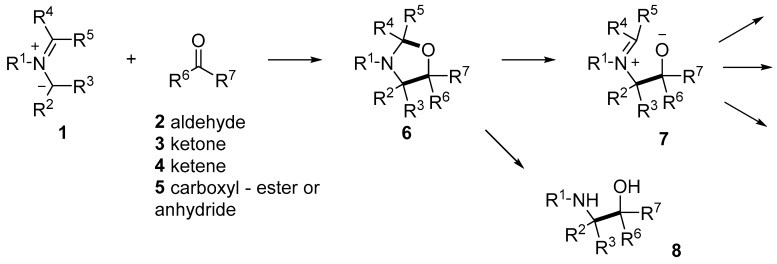
1,3-Dipolar cycloaddition reactions of azomethine ylides and carbonyl compounds to give oxazolidines and chemistry of the oxazolidines.

**Figure 1:**
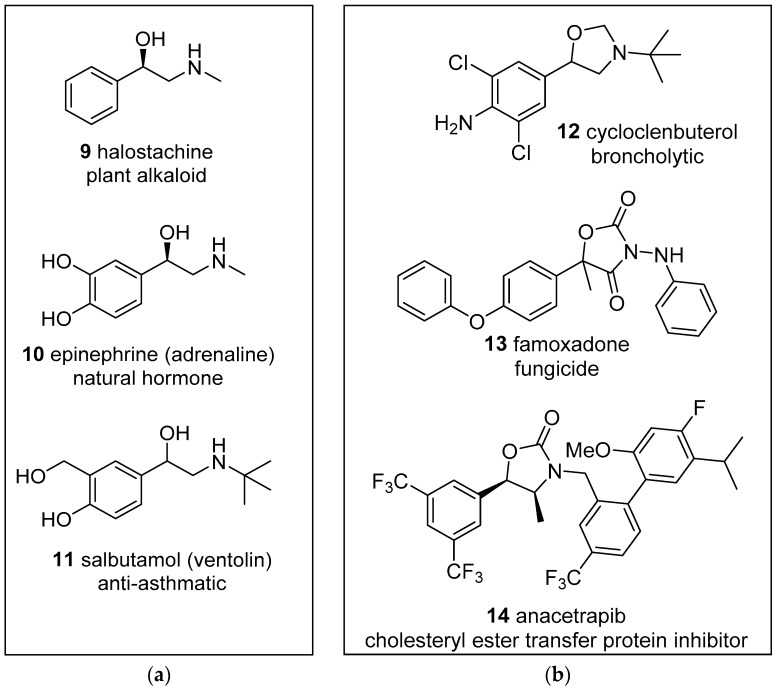
Examples of biologically active: (**a**) 1-phenyl-1-ethanol-2-amine derivatives; (**b**) oxazolidine derivatives.

There has been a resurgence of interest in reactions with carbonyl compounds, including aldehydes **2**, ketones **3**, ketenes **4** or carboxylates **5** as the heterodipolarophile, which yield oxazolidine derivatives **6** (Scheme 1). This recent interest has been due to the recognition that anhydrides such as isatoic anhydride and phthalic anhydride can act as carbonyl dipolarophiles in cycloadditions with azomethine ylides [40,41]. Additionally, the oxazolidine products **6** have a propensity to ring-open to reveal alkoxide-iminium reactive intermediates **7** which can undergo a diversity of interesting and potentially useful transformations [40,42]. Furthermore, hydrolysis of the oxazolidine products **6** leads to 1,2-ethanolamines **8**, the substructure of which can be found in a wide array of compounds of natural and synthetic origins, e.g., halostochine (**9**), epinephrine (**10**) and salbutamol (**11**) (Figure 1a) [43,44,45]. Finally, the oxazolidine ring is a core substructure of bioactive compounds of pharmaceutical and agrichemical interest, such as cycloclenbuterol (**12**), famoxadone (**13**) and anacetrapib (**14**) (Figure 1b) [46,47,48]. Herein, we provide a comprehensive review of the 1,3-dipolar cycloaddition reactions of azomethine ylides and carbonyl dipolarophiles and note interesting reactions of the oxazolidine products.

## 2. Review of Reactions of Azomethine Ylides with Carbonyl Compounds

Each section of this review refers to different types of azomethine ylide and/or different synthetic methods used to form the azomethine ylide. Within each section, examples of carbonyl dipolarophiles such as formaldehyde, aldehydes, ketones and anhydrides are provided, in that order.

### 2.1. Reactions with Ylides Formed from Ring-Opening of Aziridines

**Scheme 2:**
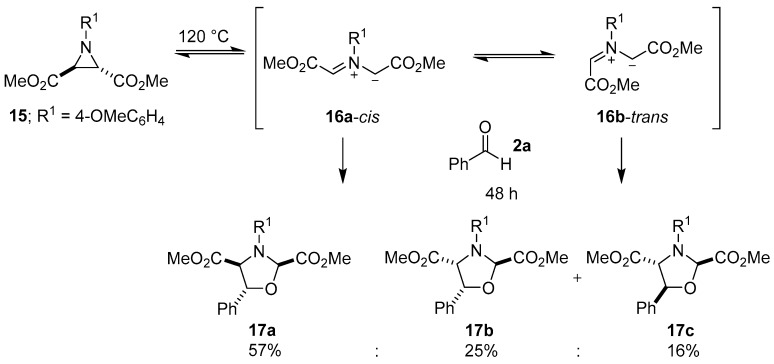
Cycloaddition of azomethine ylides **16a**-*cis* and **16b**-*trans* to benzaldehyde (**2a**).

It is fitting that Huisgen in 1967 disclosed the first 1,3-dipolar cycloaddition reaction between an azomethine ylide generated by thermal carbon-carbon bond cleavage of an aziridine ring and a carbonyl group. In this brief account, a *trans*-2,3-dimethoxycarbonyl-1-arylaziridine was heated in the presence of benzaldehyde to give a high yield of the corresponding oxazolidine as three stereoisomers [49]. A more detailed report of this work, published in 1971, described the reaction between *trans*-2,3-dimethoxycarbonyl-1-(4-methoxyphenyl)aziridine (**15**) and an excess of benzaldehyde (**2a**) to form three diasteromeric oxazolidines **17a**–**c**. When heated, *trans*-aziridine **15** underwent a conrotatory ring-opening (according to Woodward-Hoffmann theory) to generate *cis*-azomethine ylide **16a**, which primarily underwent a cycloaddition reaction with the carbonyl group of **2a** to form *cis*-2,4-oxazolidine **17a**, but also interconverted to the more stable *trans*-isomer **16b**, which then reacted with **2a** to form lesser amounts of the *trans*-2,4-oxazolidines **17b** and **17c** (Scheme 2). Aziridine **15** also underwent ring-opening and diastereoselective cycloaddition with the ketone group within diethyl ketomalonate, to predominantly form the corresponding *cis*-2,4-oxazolidine (not shown) [50].

**Scheme 3:**
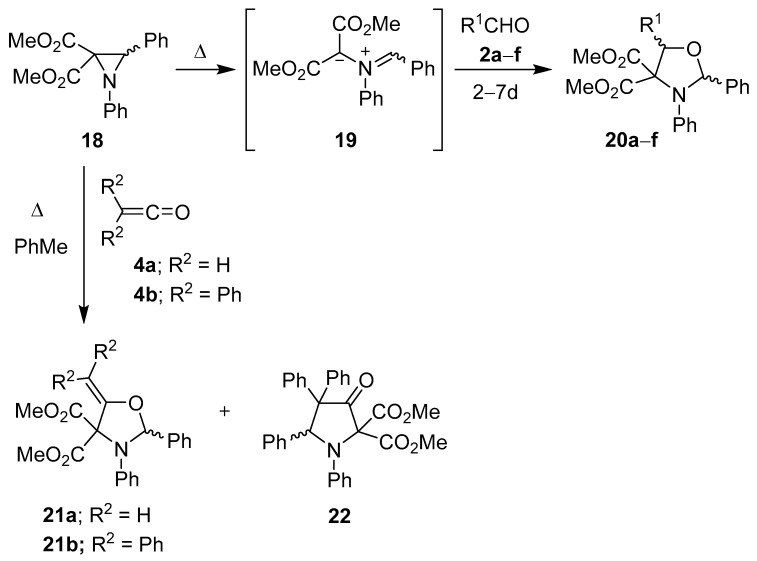
Cycloaddition of azomethine ylide **19** to aldehydes **2a**–**f** and to ketenes **4a** and **4b**.

Two years after Huisgen’s initial report, Texier and Carrié reported a number of cycloaddition reactions involving the azomethine ylide generated from the thermal ring-opening of 1,3-diphenyl-2,2-methoxycarbonylaziridine (**18**). Cycloaddition reactions of the derived ylide **19** with benzaldehyde (**2a**), acetaldehyde (**2b**), anisaldehyde (**2c**) and cinnamaldehyde (**2d**), gave the corresponding oxazolidines **20a**–**d** (as mostly one diastereoisomer), and with isobutyraldehyde (**2e**) and 2,2-diphenylacetaldehyde (**2f**) the oxazolidines **20e** and **f** were formed as single diastereoisomers (Scheme 3, Table 1) [51,52]. The same workers found that the ylide **19** added preferentially to the carbonyl bond of ketene **4a** to form oxazolidine **21a**, and to diphenylketene **4b** to form an 80:20 mixture of oxazolidine **21b** and pyrrolidone **22** (Scheme 3) [53].

**Scheme 4:**
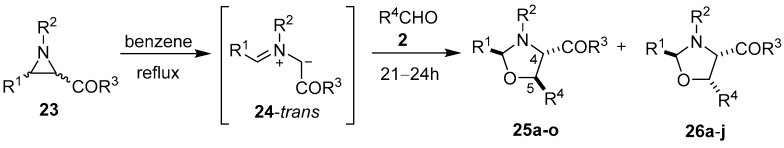
Cycloaddition of *trans*-azomethine ylide **24**, generated from 2-benzoylaziridines **23**, to aldehydes **2**.

In 1970 Lown and co-workers disclosed that a variety of 2-benzoylaziridines **23** underwent thermal [3 + 2] cycloaddition reactions with a range of aromatic aldehydes **2** to give mixtures of the diastereomeric oxazolidines **25** and **26** (Scheme 4, Table 2).

**Scheme 5:**
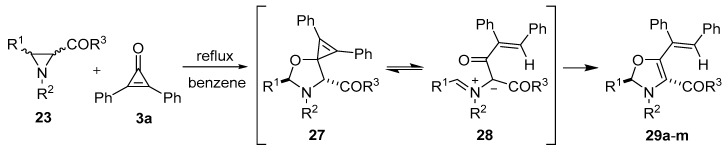
Reaction of aziridines **23** with diphenylcyclopropenone **3a** to afford 4-aroyl- and 4-acyl-4-oxazolines **29**.

When trichloroacetaldehyde (**2g**) was used as the dipolarophile, the corresponding *trans*-4,5-oxazolidines **25k**–**o** were obtained exclusively (Table 2, Entries 11–15). The orientation of the addition was confirmed by specific deuterium-labelling experiments, and the preference for the *trans*-4,5-isomer was rationalized by considering that the intermediate *cis*- and *trans*-azomethine ylides had time to equilibrate to mostly the more stable *trans*-azomethine ylide **24** prior to addition (owing to the sluggish dipolarophilic activity of the carbonyl bond). The addition of the azomethine ylide **24** to the dipolarophiles **2** then occurred in such a manner so as to predominantly form the *trans*-4,5-oxazolidines [54,55]. Two years prior, Lown’s research group also reported that azomethine ylides, derived from 3-aroyl- and 3-acylaziridines **23**, added to the carbonyl group of diphenylcyclopropenone (**3a**). This afforded intermediate oxazolidines **27** that were postulated to open to give azomethine ylides **28** and subsequently close onto the adjacent carbonyl group to afford 4-aroyl- and 4-acyl-4-oxazolines **29** (Scheme 5, Table 3) [56,57].

**Scheme 6:**
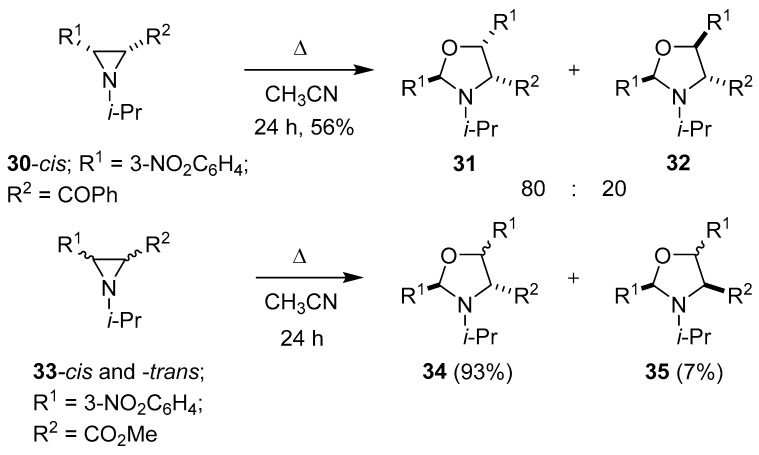
The partial fragmentation/cycloaddition reactions of aziridines **30** and **33**.

Lown and Akhtar went on to examine the thermolysis of *cis*-3-benzoylaziridine **30** in dry acetonitrile, which predominantly formed an 80:20 mixture of the corresponding stereoisomeric oxazolidines **31** and **32**, respectively, by an autocatalytic partial hydrolytic fragmentation of one molecule of aziridine **30** to give 3-nitrobenzaldehyde (**2h**) and subsequent 1,3-dipolar cycloaddition of **2h** to the azomethine ylide formed from ring opening of another molecule of aziridine **30**. A marked difference in reactivity was observed for the corresponding *trans*-aziridine isomer, which underwent mainly hydrolytic ring-cleavage processes, and only small amounts of each oxazolidine were isolated. In contrast, both *cis*- and *trans*-3-carbomethoxyaziridines **33** formed the corresponding stereoisomeric oxazolidines **34** and **35** in identical ratios and yields by an analogous partial hydrolytic fragmentation/cycloaddition process (Scheme 6) [58]. The thermolysis of an *N*-unsubstituted aziridine, dimethyl 3-[(4-nitro)phenyl]aziridine-2,2-dicarboxylate, in toluene also lead to an oxazolidine, albeit in low yield, from a partial fragmentation/cycloaddition process [59].

**Scheme 7:**
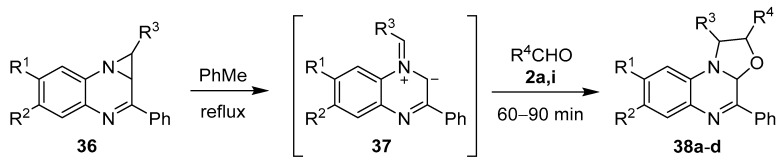
Cycloaddition of azomethine ylide **37**, generated from 1,1*a*-dihydro-1,2-diarylazirino[1,2-*a*]quinoxalines **36**, to aromatic aldehydes **2a** and **2i**.

Azomethine ylides have also been generated from aziridines fused to a quinoxoline ring system. The thermolytic ring-opening of 1,1*a*-dihydro-1,2-diarylazirino[1,2-*a*]quinoxalines **36** gave the intermediate azomethine ylides **37**, which underwent cycloadditions with benzaldehyde (**2a**) and 4-nitrobenzaldehyde (**2i**) to give oxazolo[3,2-*a*]quinoxaline derivatives **38** (Scheme 7, Table 4) [38,60].

**Scheme 8:**
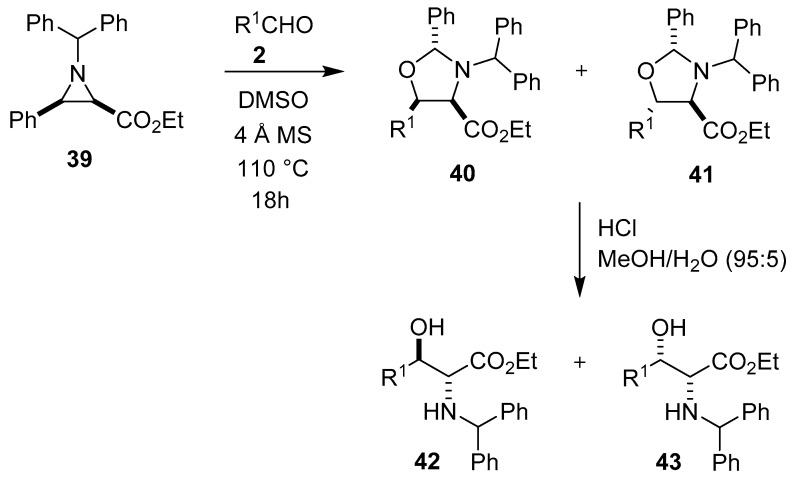
Reaction of aziridine **39** with aldehydes **2** to afford oxazolidines **40** and **41**.

The thermal electrocyclic ring-opening of benzhydryl-protected aziridine **39** gives a *cis*-azomethine ylide in equilibrium with a *trans*-azomethine ylide. The *trans*-ylide underwent a series of [3 + 2] cycloaddition reactions with mostly aromatic aldehydes **2** to form oxazolidines **40** and **41**, with the *cis*-4,5-oxazolidines **40** being the major stereoisomers isolated. In most cases, reaction mixtures comprising both oxazolidines **40** and **41** were directly hydrolyzed to the corresponding α-amino-β-hydroxy esters **42** and **43**, with the *anti*-diastereomer **42** formed as the major product (Scheme 8) [61].

**Scheme 9:**
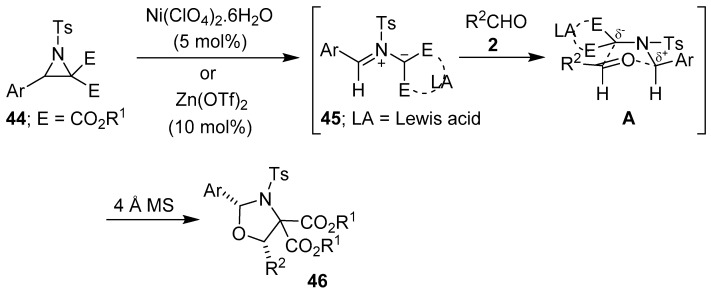
Lewis acid-promoted cycloaddition of azomethine ylides **45**, generated from aziridines **44**, to aldehydes **2**.

More recently, Zhang and co-workers have disclosed an efficient, mild and general nickel(II)-catalyzed diastereoselective [3 + 2] cycloaddition reaction of *N*-tosylaziridine 2,2-dicarboxylates **44** with aromatic aldehydes **2** to afford highly substituted 1,3-oxazolidines **46** (Scheme 9, Table 5). Presumably this process proceeds via the Lewis acid-promoted carbon-carbon bond cleavage of aziridines **44**, generating Lewis acid-coordinated azomethine ylides **45**, followed by [3 + 2]-cycloaddition reactions with the carbonyl group of aldehydes **2** via transition state **A**. The nickel-catalyzed process proved to be scalable, in contrast to the corresponding thermally-promoted process [62]. When catalyzed by Zn(OTf)_2_, this [3 + 2] cycloaddition process afforded exclusively the *cis*-2,5-disubstituted oxazolidines **46** (Scheme 9, Table 6) [63].

**Scheme 10:**
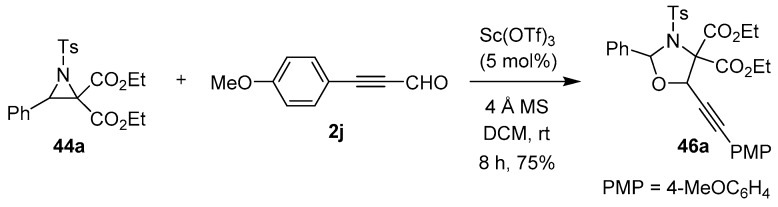
Reaction of *N*-tosylaziridine **44a** with (4-methoxyphenyl)propiolaldehyde (**2j**) to form 1,3-oxazolidine **46a**.

Zhang’s research group also reported a single example of a highly chemoselective Sc(III)-catalyzed [3 + 2] cycloaddition of the azomethine ylide derived from *N*-tosylaziridine **44a**, which reacted somewhat surprisingly with the carbonyl group rather than the alkyne group of (4-methoxyphenyl)propiolaldehyde (**2j**) to form 1,3-oxazolidine **46a** (Scheme 10) [64].

### 2.2. Reactions with Ylides Formed from Decarboxylative Condensation of Secondary α-Amino Acids and Carbonyl Compounds

**Scheme 11:**
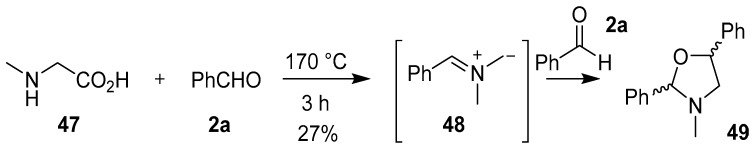
Cycloaddition of azomethine ylide **48** to benzaldehyde (**2a**).

The decarboxylative condensation reaction of secondary α-amino acids and carbonyl compounds is a convenient method to generate a wide variety of non-stabilized azomethine ylides. The first evidence for the generation of a non-stabilized azomethine ylide in this manner was reported by Rizzi in 1970. This work described the decarboxylation of sarcosine (**47**) in an excess of benzaldehyde (**2a**) to form the corresponding azomethine ylide **48**, which underwent a 1,3-dipolar cycloaddition reaction with a second molecule of benzaldehyde (**2a**) to form the oxazolidine **49**, albeit in low yield (Scheme 11). A similar reaction between sarcosine and excess benzophenone gave an array of products derived from both resonance forms of the corresponding azomethine ylide, which included a small isolable amount of an oxazolidine product (not shown) [65].

**Scheme 12:**
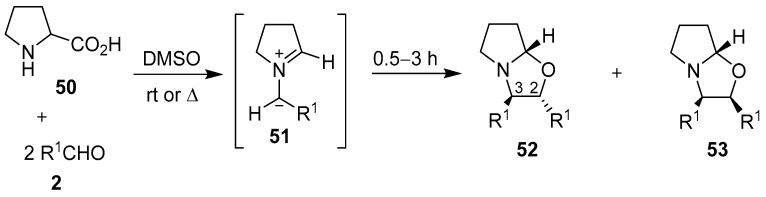
Cycloaddition of azomethine ylides **51**, generated from proline (**50**) and aromatic aldehydes **2**, to a second mole of aldehyde.

**Scheme 13:**
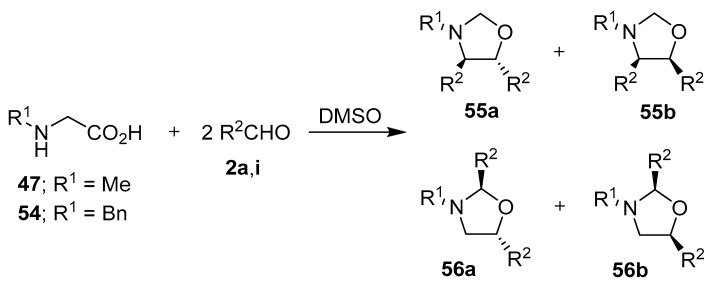
Reactions of *N*-substituted glycine derivatives **47** or **54** with aldehydes **2a** and **2i** to afford the respective 4,5- and 2,5-disubstituted-1,3-oxazolidines **55** and **56**.

Fifteen years later, Orsini and co-workers reported a study on the generation of azomethine ylides from an aldehyde-induced decarboxylation of secondary α-amino acids, and their subsequent 1,3-dipolar cycloadditions to a second mole of aldehyde to form oxazolidines [66]. A more detailed account of their work appeared in 1988, where proline (**50**) was reacted with various aromatic aldehydes **2** to afford, with complete regioselectivity, the hexahydropyrrolo[2,1-*b*]oxazoles **52** and **53**, with the *trans*-2,3-oxazolidine **52** being either the exclusive or major stereoisomer formed [67,68,69]. The stereoselectivity of the cycloaddition was governed by both the ylide stereochemistry, with the *anti*-azomethine ylide **51** being favored, and the mutual orientation of the dipole and dipolarophile (Scheme 12, Table 7). The reaction of *N*-substituted glycine derivatives **47** or **54** with aldehydes **2a** and **2i** was also investigated, with complex mixtures of the respective 4,5- and 2,5-disubstituted-1,3-oxazolidines **55** and **56** being isolated (Scheme 13, Table 8). When this method was applied to *N*-benzylalanine and aldehydes **2a** or **2i**, complex mixtures of regio- and stereoisomeric oxazolidines were also obtained.

**Scheme 14:**
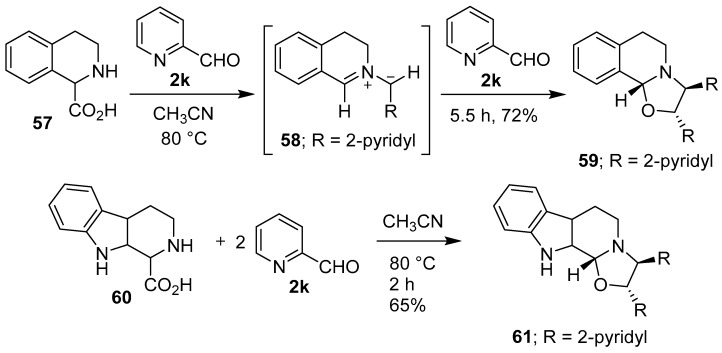
Reactions of cyclic secondary amino acids **57** and **60** with 2-pyridylcarboxaldehyde (**2k**).

**Scheme 15:**
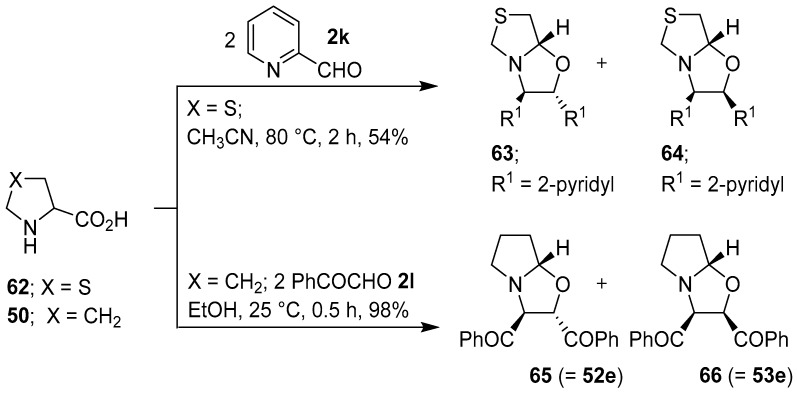
Reaction of thiazolidine-4-carboxylic acid (**62**) with 2-pyridylcarboxaldehyde (**2k**), and of proline (**50**) with phenylglyoxal (**2l**).

Grigg and co-workers found that cyclic secondary amino acid **57** reacted regio- and stereo-specifically with 2-pyridylcarboxaldehyde (**2k**), via the *anti*-azomethine ylide **58**, to give the corresponding oxazolidine **59** in good yield. In a similar manner, β-carboline **60** reacted with aldehyde **2k** to give oxazolidine **61** (Scheme 14). In further examples, thiazolidine-4-carboxylic acid (**62**) reacted regiospecifically with **2k** to give a 2:1 mixture of **63** and **64**, and proline (**50**) reacted with phenylglyoxal (**2l**) to give a 5:1 mixture of **65** and **66** (Scheme 15) [70,71]. Proline (**50**) also reacted with 2-(allyloxy)benzaldehyde to afford an azomethine ylide that underwent intermolecular cycloaddition with the aldehyde moiety of another equivalent of 2-(allyloxy)benzaldehyde to give the corresponding hexahydropyrrolo[2,1-*b*]oxazole in 51% yield rather than intramolecular cycloaddition with the unactivated internal olefin [72]. Furthermore, proline underwent a step-wise decarboxylative condensation with 2-phenylbenzaldehyde followed by cycloaddition with the same aldehyde to give the corresponding oxazolidine as a single isomer in low yield [73]. l-Proline reacted with (a) ethyl pyruvate to give the corresponding hexahydropyrrolo[2,1-*b*]oxazole derivative as a mixture of diastereomers in high yield [74]; and (b) with 4-nitrobenzaldehyde in a Ce(IV) oxide catalyzed cycloaddition reaction [75]. The reaction of sarcosine or L-proline with heteroaryl-2-carboxaldehydes afforded inseparable diastereomeric mixtures of the corresponding decarboxylative condensation-cycloaddition products [76].

**Scheme 16:**
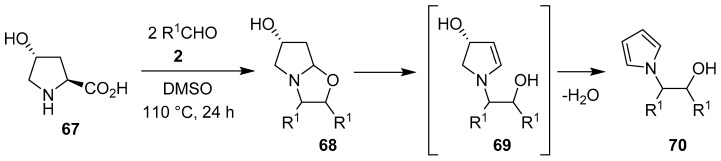
Reaction of *trans*-4-hydroxyproline (**67**) with aromatic aldehydes **2** to afford *N*-β-hydroxyethyl pyrroles **70**.

In an interesting one-pot domino process, *trans*-4-hydroxyproline (**67**) reacted with electron-deficient aromatic aldehydes **2** to give bicyclic 1,3-oxazolidines **68**, which presumably underwent ring-opening to form intermediate **69**, with subsequent dehydrative aromatization to furnish *N*-β-hydroxyethyl pyrroles **70** (Scheme 16, Table 9) [77]. In a similar fashion, indoline-2-carboxylic acid afforded the corresponding *N*-β-hydroxyethyl indoles in 33%–82% yield.

**Scheme 17:**
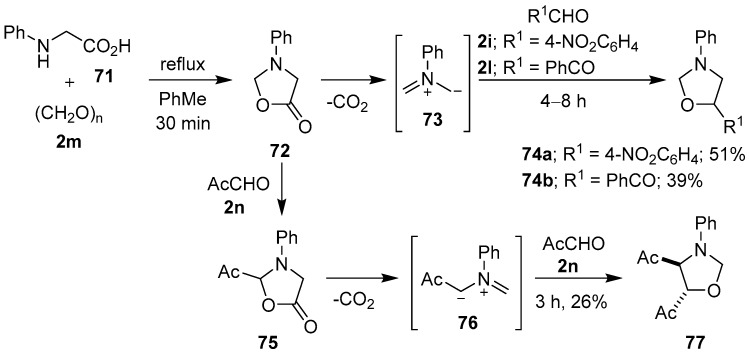
Cycloadditions of non-stabilized azomethine ylide **73** to aldehydes **2i** and **2l**, and of stabilized azomethine ylide **76** to methyl glyoxal (**2n**).

A number of research groups have provided direct evidence to support the intermediacy of azomethine ylides from the decarboxylative condensation of α-amino acids with carbonyl compounds [78,79,80,81]. Tsuge and co-workers briefly heated *N*-phenylglycine (**71**) at reflux with paraformaldehyde (**2m**) to produce isolable 3-phenyl-5-oxazolidinone (**72**). When oxazolidinone **72** was further heated, decarboxylation occurred to give non-stabilized azomethine ylide **73**, which underwent cycloaddition with 4-nitrobenzaldehyde (**2i**) or phenylglyoxal (**2l**) to afford the respective oxazolidines **74** in modest yields. Oxazolidinone **72** also underwent an acetal-exchange reaction with methyl glyoxal (**2n**) to afford acetyl-substituted 5-oxazolidinone **75**. This compound then underwent decarboxylation to generate stabilized azomethine ylide **76**, which was captured by another equivalent of aldehyde **2n** to give cycloadduct **77** (Scheme 17) [80].

**Scheme 18:**
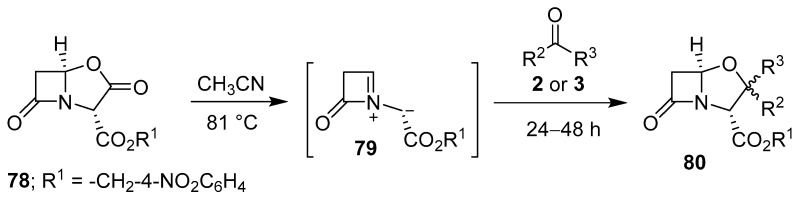
Cycloaddition of azomethine ylide **79** to aldehydes **2** or ketones **3**.

In a related decarboxylative process, thermolysis of lactam-based oxazolidinone **78** generated the corresponding four-membered lactam incorporating an azomethine ylide **79**, which underwent cyloadditions with aldehydes **2** and ketones **3** to furnish mostly 1:1 diastereomeric mixtures of oxapenam derivatives **80** in low to moderate yields (Scheme 18, Table 10) [82].

**Scheme 19:**
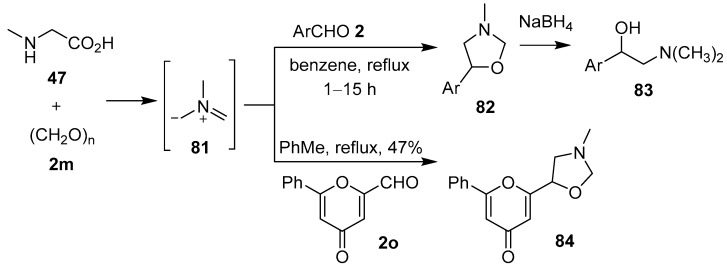
Cycloaddition of azomethine ylide **81** to aromatic aldehydes **2** or pyrone aldehyde **2o**.

The non-stabilized azomethine ylide **81**, derived in situ from sarcosine (**47**) and paraformaldehyde (**2m**), underwent 1,3-dipolar cycloaddition reactions with various aromatic aldehydes **2** to form the corresponding 5-aryl-3-methyloxazolidines **82** (Scheme 19, Table 11). Subsequent reductive ring-opening of the cycloadducts afforded 1-aryl-2-dimethylaminoethanols **83** [83]. This work was predicated on the observation that sarcosine (**47**), paraformaldehyde (**2m**) and pyrone aldehyde **2o** reacted to form *N*-methyloxazolidine **84** [84]. Azomethine ylide **81**, formed in this way, also underwent cycloaddition reactions with 2-chloro-3-quinolinecarboxaldehydes to afford the corresponding 3-quinolyl-1,3-oxazolones [85], and in an internal competition reaction with 3-formyl-2*H*-chromene to furnish a 2.8:1 mixture of the corresponding benzo[3,4-*c*]pyrrolidine and oxazolidine cycloadducts, respectively [86].

**Scheme 20:**
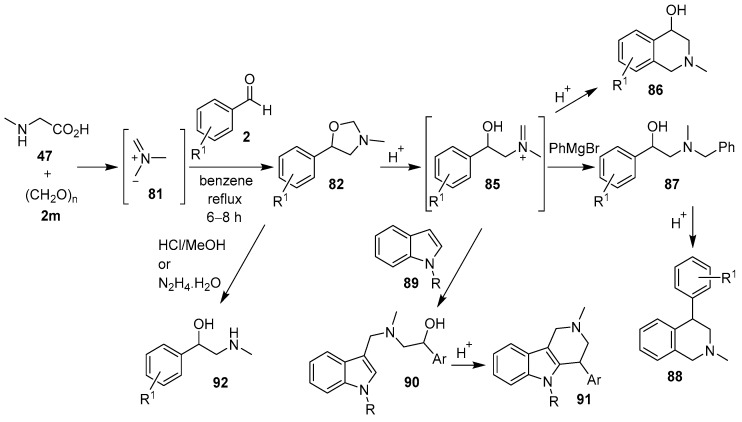
Cycloaddition of azomethine ylide **81** to aromatic aldehydes **2**, and the subsequent products derived from ring-opening reactions of 5-aryloxazolidine **82**.

In a series of recent publications, Moshkin, Sosnovskikh and co-workers have also utilized the azomethine ylide **81** in 1,3-dipolar cycloaddition reactions with the carbonyl bond of various aromatic aldehydes **2**. The synthetic utility of the corresponding 5-aryloxazolidine products was explored, and in a series of acid-catalyzed ring-opening reactions via iminium ion **85**, a number of heterocycles and 2-amino-1-arylethanols were formed (Scheme 20). Thus, the 5-aryloxazolidines **82** underwent (a) acid-catalyzed rearrangement into 2-methyl-1,2,3,4-tetrahydroisoquinolin-4-ols **86** [42]; (b) reaction with arylmagnesium bromides to produce *N*-benzyl-β-hydroxyphenethylamines **87**, which underwent subsequent acid-catalyzed cyclization to give 4-aryl-1,2,3,4-tetrahydro-isoquinolines **88** [87]; and (c) reaction with indoles **89** to afford 2-(indol-3-ylmethylamino)-1-aryl ethanols **90**, which underwent acid-catalyzed cyclization to yield 4-aryl-2-methyl-1,2,3,4-tetrahydro-β-carbolines **91** [88]. Alternatively, oxazolidines **82** could be readily hydrolyzed to form 2-(alkylamino)-1-arylethanols **92** by treatment with acid or hydrazine hydrate [89].

**Scheme 21:**
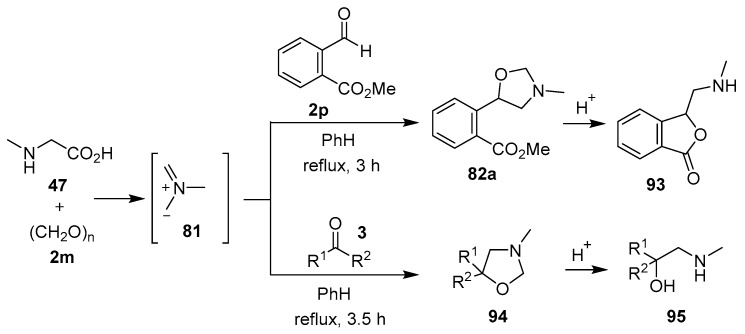
Cycloaddition of azomethine ylide **81** to aldehyde **2p** and ketones **3**, and the subsequent oxazolidine ring-opened products.

The sarcosine/formaldehyde derived azomethine ylide **81** also reacted with (a) aromatic aldehydes bearing an ester group such as **2p**, which formed the corresponding oxazolidine **82a**, and underwent a subsequent demethylenation/rearrangement process to form (aminomethyl)lactone **93** [90]; and (b) a variety of aromatic ketones **3** to give the 5,5-diaryloxazolidines **94** (Scheme 21, Table 12), which underwent hydrolysis to afford 2-alkylaminoethanols **95** (Scheme 21) [91].

**Scheme 22:**
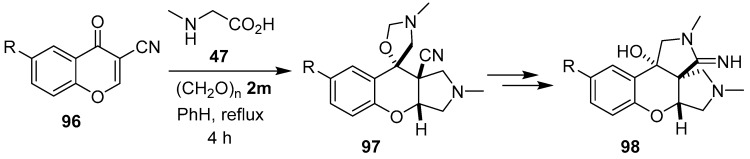
Dual cycloaddition of azomethine ylide **81** to 3-cyanochromones **96**.

The same researchers found that an excess of the sarcosine/paraformaldehyde derived azomethine ylide **81** participated in a dual 1,3-dipolar cycloaddition reaction with 3-cyanochromones **96** at both the double bond and the carbonyl group to give diastereoselectively tetrahydro-1*H*-spiro[chromeno[2,3-*c*]pyrrol-9,5′-oxazolidine]-9*a*-carbonitriles **97**, which subsequently underwent hydrolysis and cyclization of the amino group onto the nitrile to give hexahydrochromeno[2,3-c:3,4-c′]dipyrroles **98** (Scheme 22, Table 13) [92,93].

**Scheme 23:**
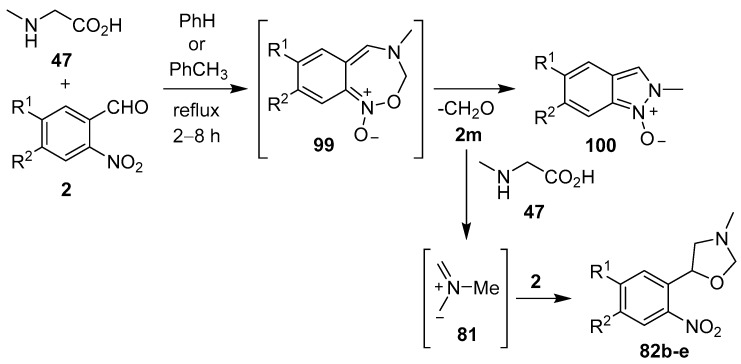
Reaction of sarcosine (**47**) with 2-nitrobenzaldehydes **2**.

Azomethine ylides, generated from sarcosine (**47**) and substituted 2-nitrobenzaldehydes **2**, underwent 1,7-electrocylization onto the adjacent nitro group to give unstable benz-1,2,6-oxadiazepines **99**. Subsequent ring-contraction afforded mixtures of indazole-*N*-oxides **100** and 3-methyl-5-aryloxazolidines **82b**–**e**. It was postulated that the oxazolidine products were derived from the paraformaldehyde (**2m**) eliminated during this process, that reacted with excess sarcosine (**47**) to generate azomethine ylide **81**, which underwent cycloaddition to another molecule of a 2-nitrobenzaldehyde derivative **2** (Scheme 23, Table 14) [94,95].

**Scheme 24:**
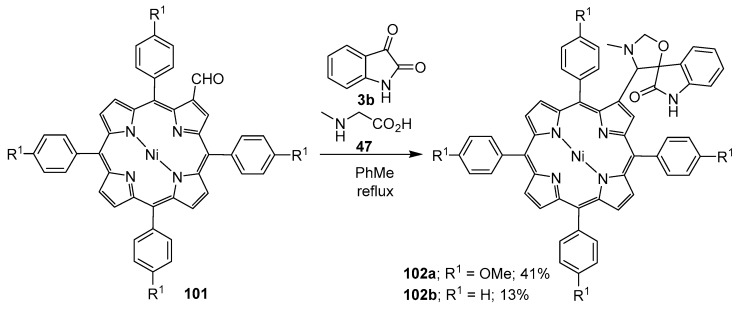
Cycloaddition of azomethine ylides generated from sarcosine (**47**) and porphyrins **101** to isatin (**3b**).

Azomethine ylides generated from sarcosine (**47**) and Ni(II) β-formyl-*meso*-tetraphenyl-porphyrins **101** underwent regioselective cycloaddition reactions with isatin (**3b**) to furnish the corresponding spiroporphyrin derivatives **102** in modest yields (Scheme 24) [96].

**Scheme 25:**
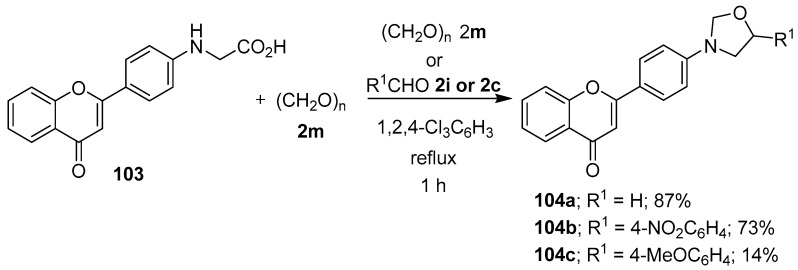
Cycloaddition of the azomethine ylide generated from *N*-[4-(chromon-2-yl)phenyl]glycine (**103**) and paraformaldehyde (**2m**) to a second molecule of paraformaldehyde (**2m**) or to aldehydes **2i** or **2c**.

The azomethine ylide generated in situ from the reaction of *N*-[4-(chromon-2-yl)phenyl]glycine (**103**) and paraformaldehyde (**2m**) was trapped with either a molecule of paraformaldehyde (**2m**) or the aromatic aldehydes **2i** or **2c** to afford the corresponding flavone-oxazolidine dyads **104** (Scheme 25) [97]. Minor amounts of the corresponding oxazolidine cycloadducts have also been isolated as side products from reactions involving (a) sarcosine, phenylglyoxal and *N*-ethylmaleimide [98]; and (b) (*S*)-(−)-2-azetidinecarboxylic acid, phenylglyoxal and dimethyl fumarate [99].

**Scheme 26:**
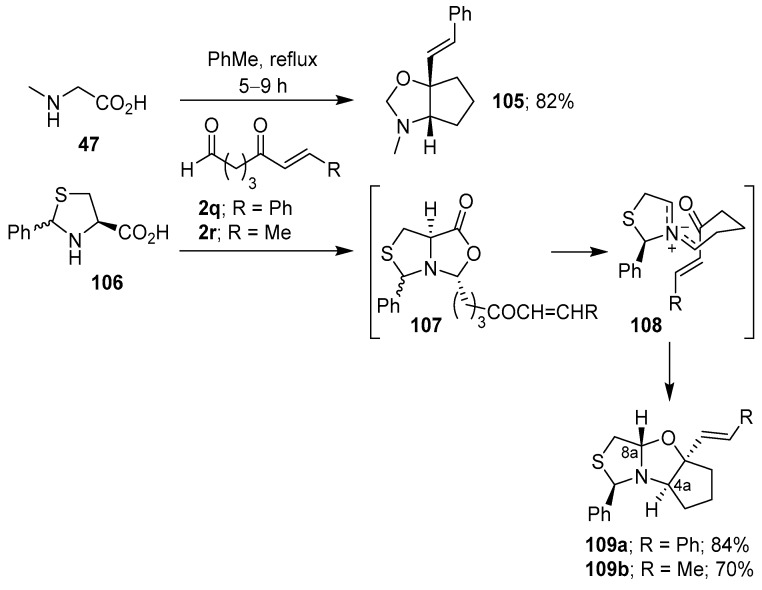
Intramolecular cycloaddition of the non-stabilized azomethine ylide generated from the condensation of α-amino acids with aldehydes bearing a tethered aldehyde.

An intramolecular variant that involves the generation of non-stabilized azomethine ylides from the condensation of α-amino acids with aldehydes bearing a tethered carbonyl group has also been reported. Thus, the azomethine ylide generated from sarcosine (**47**) and 5-oxo-7-phenyl-6-heptenal (**2q**), reacted exclusively at the internal carbonyl moiety to produce regio- and stereoselectively cycloadduct **105** in 82% yield. Similarly, 2-phenyl-4-thiazolidinecarboxylic acid (**106**) reacted with **2q** and **2r** to produce **109a** and **109b**, respectively, as single stereoisomers in good yields. The *trans*-configuration between 4a-H and 8a-H was due to the selective participation of the *Z*,*E*-ylidic forms **108**, which was presumably derived from the stereospecific decarboxylation of the more thermodynamically stable bicyclic lactones **107** (Scheme 26) [100].

**Scheme 27:**
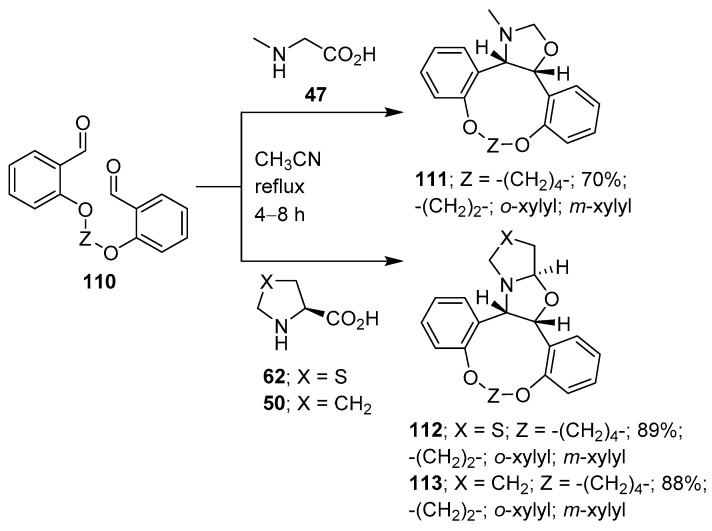
Intramolecular cycloaddition of azomethine ylides generated from dialdehyde **110** and sarcosine (**47**), thiazolidine-4-carboxylic acid (**62**) or L-proline (**50**).

Further examples of this type of intramolecular 1,3-dipolar cycloaddition process include the reaction of intermediate azomethine ylides derived from dialdehyde **110** and (a) sarcosine (**47**), which resulted in the stereoselective formation of *cis*-fused oxazolidine-grafted macrocycles **111**; (b) thiazolidine-4-carboxylic acid (**62**) to form tetrahydrooxazolothiazoles **112**; and (c) L-proline (**50**) to give hexahydropyrrolooxazole grafted macrocycles **113** (Scheme 27) [101].

### 2.3. Reactions of Ylides Formed by Condensation of Amines with Carbonyl or 1,2-Dicarbonyl Compounds

**Scheme 28:**
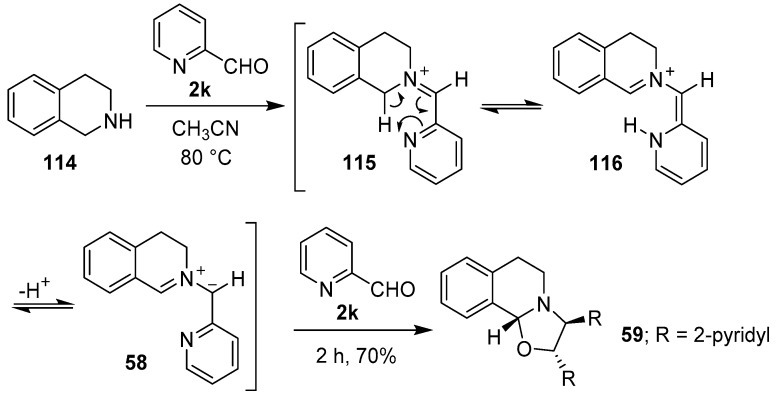
Cycloaddition of azomethine ylide **58** to 2-pyridaldehyde (**2k**).

In 1986, Grigg and co-workers reported that azomethine ylides could be derived from iminium ions formed between primary or secondary amines and bifunctional carbonyl compounds. Thus, tetrahydroisoquinoline (**114**) and 2-pyridaldehyde (**2k**) reacted to form iminium ion **115**, which was postulated to undergo a formal sigmatropic 1,5-H shift to generate intermediate **116**, and subsequently regio- and stereospecifically, the *anti*-azomethine ylide **58**. Cycloaddition of the derived 1,3-dipole with another molecule of the aldehyde afforded oxazolidine **59** (Scheme 28). In a similar manner, β-carboline reacted with the aldehyde **2k** to give oxazolidine **61** in 64% yield [102,103].

**Scheme 29:**
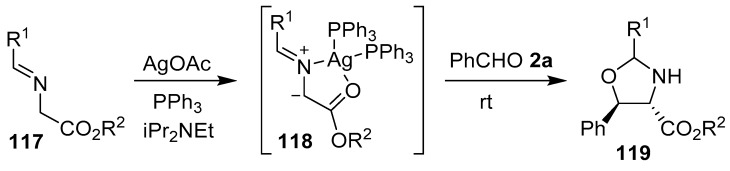
Cycloaddition of *N*-metalated azomethine ylides **118**, generated from α-iminoesters **117**, to benzaldehyde (**2a**).

To prepare *N*-unsubstituted oxazolidines, a hydrogen or a metal atom must be attached to the nitrogen atom of the azomethine ylide. *N*-Metalated ylides of this type have most often been generated by treatment of an imine bearing an electron withdrawing group with a metal salt, which coordinates with the imine nitrogen to activate the substrate towards deprotonation by an added base. Thus, activation of aromatic aldehyde-derived α-iminoesters **117** by a Ag(I)/PPh_3_ catalyst generated the *N*-metalated azomethine ylides **118**, which underwent cycloadditions with benzaldehyde **2a** to yield predominantly 4,5-*trans* oxazolidines **119** (Scheme 29, Table 15). Subsequent hydrolysis of the oxazolidine cycloadducts furnished the corresponding *syn*-β-aryl-β-hydroxy-α-amino esters [104].

**Scheme 30:**
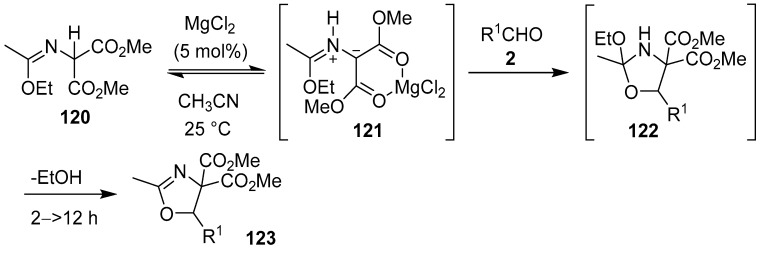
Cycloaddition of azomethine ylide **121**, generated from malonic imidate **120** via 1,2-prototropy, to aldehydes **2**.

In a Lewis acid-catalyzed process, malonic imidate **120** was postulated to chelate to MgCl_2_ to form intermediate azomethine ylide **121** via 1,2-prototropy. The metal-coordinated ylide was subsequently trapped by various aldehydes **2** to form the corresponding oxazolidines **122**, which underwent spontaneous loss of ethanol to afford oxazolines **123** (Scheme 30, Table 16) [105].

**Scheme 31:**
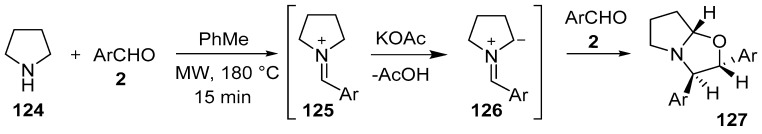
Cycloaddition of azomethine ylide **126**, generated from pyrrolidine (**124**) and aromatic aldehydes **2**, to a second molecule of aldehyde **2**.

In a metal-free, base promoted process under microwave irradiation, pyrrolidine (**124**) reacted with aromatic aldehydes **2** to form the corresponding iminium ions **125**, which were presumably transformed into azomethine ylides **126** via α-deprotonation by acetate anion. The [3 + 2] cycloaddition reaction between the derived azomethine ylide and another molecule of aldehyde afforded the corresponding hexahydropyrrolo[2,1-*b*]oxazoles **127**, in a diastereoselective fashion (Scheme 31, Table 17). Piperidine reacted in an analogous fashion. This process can be considered as a direct functionalization of an sp^3^ C-H bond of an unfunctionalized cyclic amine [106]. In a similar manner, but under milder conditions, 1,2,3,4-tetrahydroisoquinoline and tetrahydro-β-carboline reacted with aromatic aldehydes to furnish the corresponding oxazolo[2,3-*a*]isoquinolines as single diastereomers [107].

### 2.4. Reactions of Ylides from the Condensation of Secondary Alpha Amino Acid Esters with Carbonyl Compounds

**Scheme 32:**
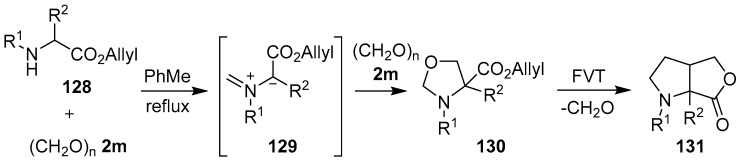
Cycloaddition of azomethine ylide **129**, generated from allylic α-amino esters **128** and paraformaldehyde (**2m**), to a second molecule of paraformaldehyde (**2m**), and subsequent FVT-induced cycloreversion-intramolecular cycloaddition.

A simple approach to generate stabilized azomethine ylides is via the reaction of a secondary amine, bearing an α-carboxylic ester functionality, with an aldehyde, and the subsequent deprotonation of the resultant iminium ion. The first example of an azomethine ylide being generated in this manner and participating in a 1,3-dipolar cycloaddition with a carbonyl bond to form an oxazolidine ring, was published by Joucla and co-workers in 1987 [108]. In this report, the condensation of allylic α-amino esters **128** with paraformaldehyde (**2m**) presumably gave rise to the corresponding azomethine ylides **129**, which underwent intermolecular cycloadditions with a second equivalent of paraformaldehyde (**2m**) to afford oxazolidines **130** in good yields. When these oxazolidines were subjected to flash vacuum thermolysis (FVT), a cycloreversion process occurred to regenerate azomethine ylide **129**, which underwent an intramolecular cycloaddition reaction with the adjacent alkene to furnish pyrrolidines **131** (Scheme 32, Table 18).

**Scheme 33:**
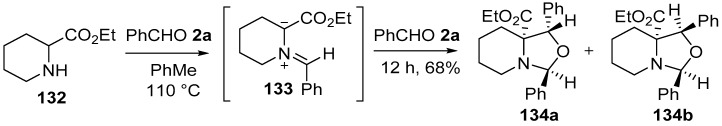
Cycloaddition of azomethine ylide **133**, generated from pipecolic acid ethyl ester (**132**) and benzaldehyde (**2a**), to a second molecule of benzaldehyde (**2a**).

Around the same time, Joucla and co-workers reported that the azomethine ylide **133**, prepared in situ from the condensation of pipecolic acid ethyl ester (**132**) and benzaldehyde (**2a**) underwent a 1,3-dipolar cycloaddition with a second molecule of benzaldehyde (**2a**) to form a 70:30 mixture of the oxaindolizidines **134a** and **134b**, respectively (Scheme 33) [109]. The stereoselectivity of the cycloaddition reaction was governed by both the conformation of the ylide, and the orientation at which the dipole and dipolarophile approached. Furthermore, the same workers disclosed the synthesis of a number of oxazolidines derived from reactions between proline, sarcosine and *N*-benzylglycinate methyl esters and two molar equivalents of formaldehyde or benzaldehyde [110].

**Scheme 34:**
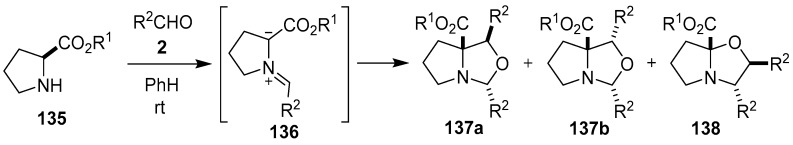
Cycloaddition of azomethine ylide **136**, generated from L-proline alkyl esters **135** and aromatic aldehydes **2**, to a second molecule of aldehyde **2**.

The reaction between l-proline alkyl esters **135** and aromatic aldehydes **2** generated the azomethine ylides **136**, which were trapped by a second aldehyde molecule to give mostly mixtures of the oxapyrrolizidines **137a**, **137b** and **138** (Scheme 34, Table 19). The oxapyrrolizidines were prone to cycloreversion to regenerate azomethine ylides **136**, and in the presence of a range of conjugated nitroolefins underwent cycloaddition to afford the corresponding pyrrolizidines [111]. A number of reactions of *N*-alkylated glycine ethyl esters and paraformaldehyde have also been reported, which were conducted neat and under microwave irradiation, to afford the corresponding oxazolidines in moderate yield [112]. During a reaction involving sarcosine methyl ester, formaldehyde and *N*-ethyl maleimide, the azomethine ylide generated from sarcosine methyl ester and formaldehyde was trapped by a second molecule of formaldehyde to give very small amounts of the corresponding oxazolidine cycloadduct [98].

**Scheme 35:**
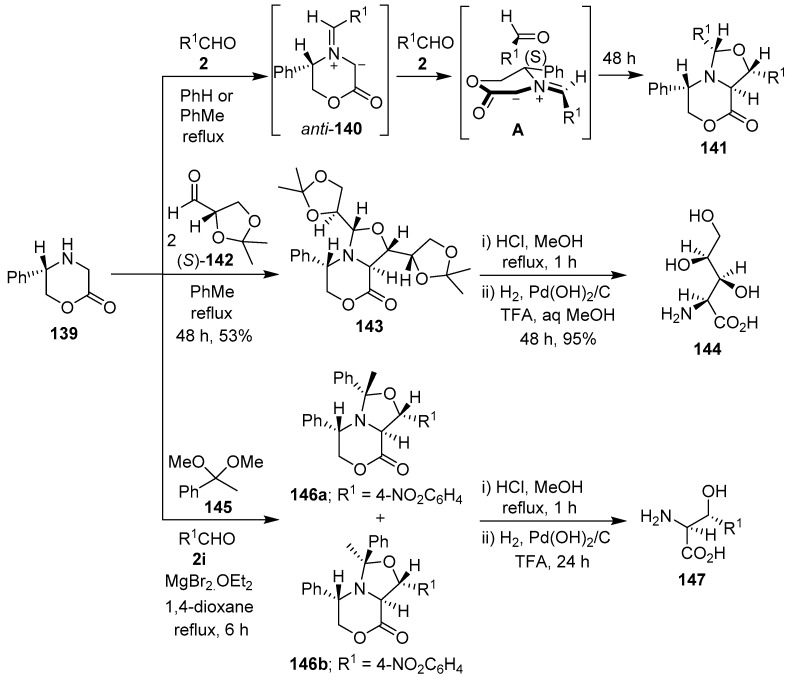
Synthesis of bicyclic oxazolidines via cycloaddition reactions of azomethine ylides, derived from 5-(*S*)-phenylmorpholine-2-one (**139**) and aldehydes **2**, **142** or acetophenone dimethyl acetal (**145**), to a second equivalent of the same or a different aldehyde.

Harwood and co-workers have reported the diastereoselective synthesis of bicyclic oxazolidines via 1,3-dipolar cycloaddition reactions of azomethine ylides derived from the condensation of 5-(*S*)-phenylmorpholine-2-one (**139**) with aldehydes **2**, and their subsequent trapping by the carbonyl bond of a second equivalent of the aldehyde **2**. Thus, chiral morpholinone **139** reacted with aldehydes **2** to generate stabilized *anti*-azomethine ylides **140**, which underwent highly diastereocontrolled cycloadditions with a second aldehyde **2** to afford cycloadducts **141**. The high stereocontrol was rationalized by cycloaddition mode **A** involving the aldehyde dipolarophile approaching the least hindered side of the *anti*-azomethine ylide, and from the face opposite the 5-phenyl substituent (Scheme 35, Table 20). Subsequent hydrogenolysis of cycloadducts **141** formed the corresponding homochiral β-hydroxy-α-amino acids [113,114,115]. When morpholinone **139** was condensed with two equivalents of (*S*)-glyceraldehyde acetonide **142**, a cycloaddition occurred that was diastereochemically ‘matched’ in both the ylide generation and trapping steps such that cycloadduct **143** was formed exclusively. Sequential hydrolysis and hydrogenolysis of cycloadduct **143** afforded polyoxamic acid **144** in an enantiomerically pure fashion (Scheme 35). In the same manner, the opposite enantiomer of polyoxamic acid was obtained by reacting (*R*)-**139** with (*R*)-**142**, followed by sequential degradation of the cycloadduct [116]. Furthermore, a chiral azomethine ylide, derived from morpholinone **139** and acetophenone dimethyl acetal (**145**), underwent Lewis acid-promoted cycloaddition with 4-nitrobenzaldehyde (**2i**) to afford *anti-exo* **146a** and *syn-exo* **146b** cycloadducts in 42% and 18%, respectively. Hydrogenolysis of either cycloadduct afforded the same product, (2*S*,3*R*)-2-amino-3-hydroxy-3-(4-aminophenyl)propanoic acid (**147**) (Scheme 35), which confirmed that **146a** and **146b** were epimeric at C-9, the stereochemistry was retained at C-7, and that both *syn*- and *anti*-ylides were involved in the cycloaddition [115,117]. Having developed this methodology, Harwood’s research group also prepared enantiopure long chain *threo*-2-amino-3-hydroxyesters via chiral azomethine ylides derived from the reaction of 5-(*R*)-phenylmorpholine-2-one with long chain aldehydes, and their subsequent trapping with a second equivalent of aldehyde [118].

**Scheme 36:**
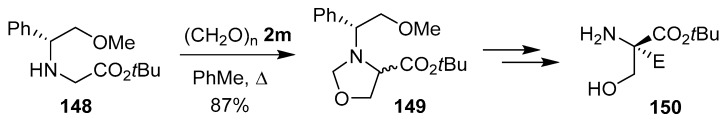
Reaction of glycine derivative **148** and paraformaldehyde (**2m**) to afford oxazolidine **149**.

Oxazolidine **149**, containing a chiral *N*-benzyl protecting group, was prepared as a 1:1 diastereomeric mixture from the reaction of glycine derivative **148** and paraformaldehyde (**2m**). Sequential diastereoselective functionalization of the corresponding oxazolidine ester enolate, then hydrolysis and hydrogenolysis afforded enantiomerically pure quaternary serine esters **150** (Scheme 36) [119].

**Scheme 37:**
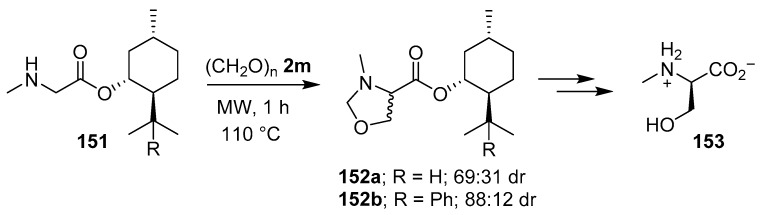
Reaction of sarcosine esters **151a** and **151b** with paraformaldehyde (**2m**) to afford oxazolidines **152a** and **152b**, respectively.

When sarcosine (−)-menthyl or (−)-8-phenylmenthyl esters, **151a** and **151b** respectively, were reacted with paraformaldehyde (**2m**) cycloaddition occurred in a diastereoselective fashion to give the respective oxazolidines **152a** and **152b**, which were exhaustively hydrolyzed to form predominantly *N*-methyl-d-serine (**153**) (Scheme 37) [120].

### 2.5. Reactions with Ylides Formed from Silylmethylamines and Related Reagents

A wide range of azomethine ylides generated from silylmethylamine reagents have been explored in reactions with carbonyl dipolarophiles.

#### 2.5.1. Desilylation of (Trimethylsilylmethyl)iminium Ions

**Scheme 38:**
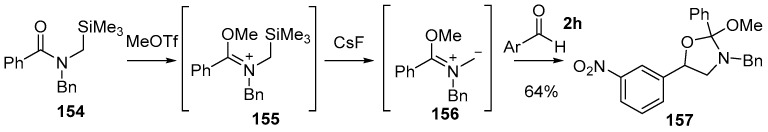
Cycloaddition of an azomethine ylide, generated from desilylation of a *N*-(trimethylsilylmethyl)-immonium ion, with 3-nitrobenzaldehyde (**2h**) (Ar = 3-NO_2_-C_6_H_3_).

Padwa demonstrated that non-stabilized azomethine ylides formed by desilylation of *N*-(trimethylsilylmethyl)-immonium ions, a process initially developed by Vedejs [121], react with aldehydes **2** [122]. The reaction of *N*-(trimethylsilylmethyl)benzamide **154** with methyl triflate afforded the trimethylsilylmethyl iminium ion **155**, which on treatment with CsF produced azomethine ylide **156**, and in the presence of 3-nitrobenzaldehyde (**2h**) afforded cycloadduct **157** as the exclusive product, indicating a highly regioselective process (Scheme 38). The regioselectivity was determined by a combination of spectroscopic analyses and chemical transformation into a known derivative, however, no comment was made about the stereoselectivity. The exclusive formation of the isolated regioisomer was rationalised to be the result of the union of the larger azomethine ylide HOMO coefficient on the unsubstituted carbon of the ylide **156** with that of the larger dipolarophile LUMO coefficient on the carbon atom of the carbonyl group of the aldehyde **2h**.

**Scheme 39:**
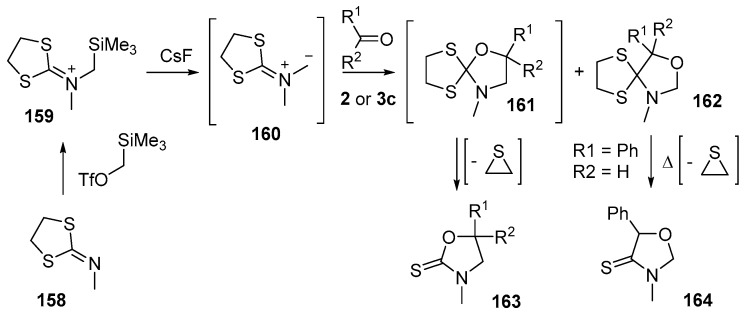
Cycloaddition of dithiolane-isocyanate immimium methylides **160** with aldehydes **2** and benzophenone (**3c**).

Dithiolane-isocyanate imminium methylides are interesting azomethine ylides that undergo efficient and regioselective cycloaddition to carbonyl compounds [123]. The azomethine ylide **160**, produced from CsF-promoted desilylation of iminodithiolane salt **159** (in turn produced by the reaction of the readily available *N*-methylimino dithiolane (**158**) with trimethylsilylmethyl triflate), reacted with aldehydes **2** or benzophenone (**3c**) to give a mixture of regioisomeric cycloadducts **161** and **162** (Scheme 39, Table 21). While the minor adducts **162** could be isolated by silica chromatography, the major adducts **161** decomposed under these conditions, with loss of thiirane, to afford the isolated thiolactam **163**. When the minor benzaldehyde cycloadduct **162a** was heated in refluxing toluene for 48 h, it underwent an analogous decomposition to give thiolactam **164** in 48% yield. The preferred regioselectivity of the cycloaddition was thought to be a result of the union of the larger LUMO coefficient on the carbon of the carbonyl group with the larger HOMO coefficient on the unsaturated centre attached to the sulfur atoms of the dithiolane ring. Interestingly, it was noted that the cycloaddition only occurred with conjugated carbonyl compounds. For attempts at cycloaddition using simple non-conjugated aldehydes and ketones, e.g., acetone, cyclohexanone and butyraldehyde, no reaction was observed.

In a related study, reaction of azomethine ylide **160** with α,β-unsaturated ketones produced mainly cycloadducts of the carbon-carbon double bond, with small quantities (5%) of the ketone cycloadduct also isolated [124].

**Scheme 40:**
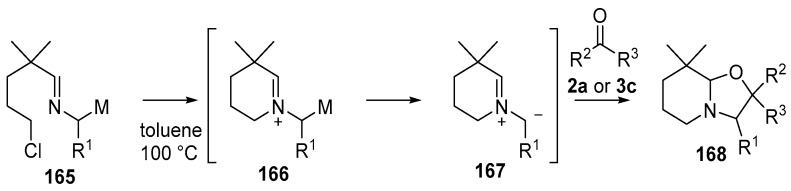
Cycloaddition of azomethine ylides, generated by an *N*-alkylation/demtallation ccascade, to aldehyde (**2a**) and benzophenone (**3c**).

Non-stabilized azomethine ylides **167** were generated from (2-azaallyl)stannanes **165a** or **b** or (2-azaallyl)silane **165c** by an intramolecular *N*-alkylation/demetallation cascade [125,126]. The ylides **167** underwent reaction with a range of dipolarophiles including with benzaldehyde (**2a**) and benzophenone (**3c**) to afford bicyclic oxazolidines **168a**–**c** (Scheme 40, Table 22).

#### 2.5.2. Desilylation of α-Substituted Methyl(trimethylsilylmethyl)amines

**Scheme 41:**
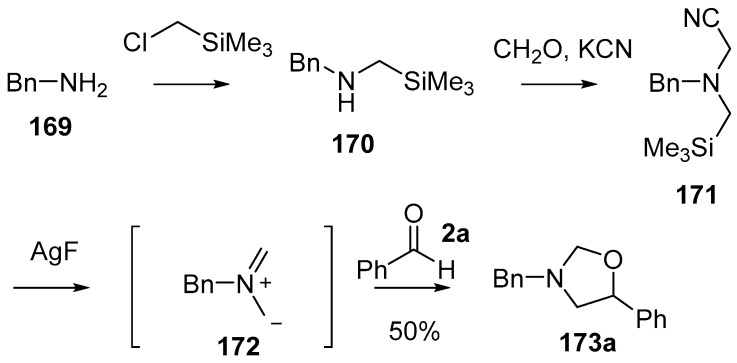
Cycloaddition of a non-stabilized ylide, derived from cyanomethylamine reagent **171**, with benzaldehyde (**2a**).

A series of α-substituted methyl(trimethylsilylmethyl)amine reagents were developed and shown to act as azomethine ylide precursors, adding to a range of dipolarophiles including carbonyl compounds. The first of these reagents to be explored in carbonyl cycloadditions was the cyanomethylamine reagent **171** [127] which was efficiently prepared from benzylamine (**169**) by firstly reacting with (chloromethyl)trimethylsilane and then formaldehyde and KCN (Scheme 41). Treatment of reagent **171** with AgF produced the non-stabilized azomethine ylide **172**, which was trapped with a range of dipolarophiles including benzaldehyde (**2a**) which produced oxazolidine **173a**, isolated in 50% yield (Scheme 41).

**Scheme 42:**
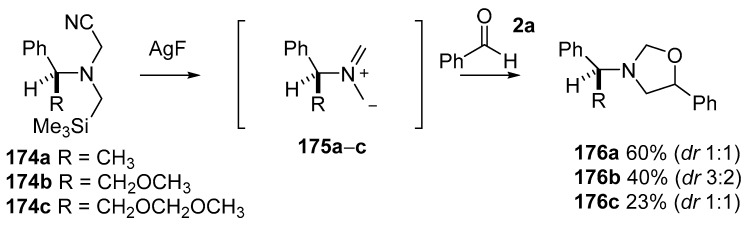
Cycloaddition of chiral non-stabilized ylides, derived from cyanomethylamine reagents **175**, with benzaldehyde (**2a**).

A series of chiral cyanoaminosilanes **174a**–**c** were produced, using the same methods as used to prepare **171**, to explore potential for asymmetric cycloaddition reactions [127]. Although cycloaddition reactions between the in situ produced ylides **175a**–**c** and benzaldehyde (**2a**) did occur to give oxazolidines **176a**–**c** respectively, little if any diastereoselectivity was observed for this carbonyl dipolarophile (Scheme 42). In contrast, improved and at times promising diastereoselectivity was observed for olefinic dipolarophiles.

**Scheme 43:**
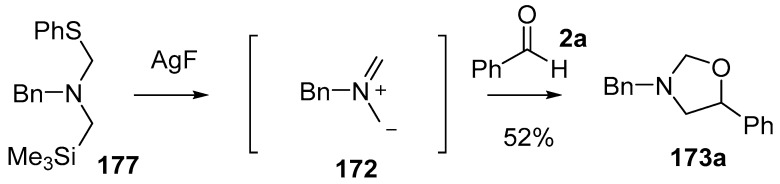
Cycloaddition of non-stabilized ylide **172**, derived from phenylthiomethylamine reagent **177**, with benzaldehyde (**2a**).

The related phenylthio-substituted amine **177** was produced from benzylamine by reacting with chloromethyltrimethylsilane followed by paraformaldehyde and thiophenol [128]. In a similar way to the cyanoaminosilane reagents **171** and **174**, when treated with AgF, reagent **177** released non-stabilized azomethine ylide **172** and the ylide formed in this way showed utility in cycloaddition reactions with a range of dipolarophiles including benzaldehyde (**2a**), in which case oxazolidine **173a** was produced in 52% yield (Scheme 43).

**Scheme 44:**
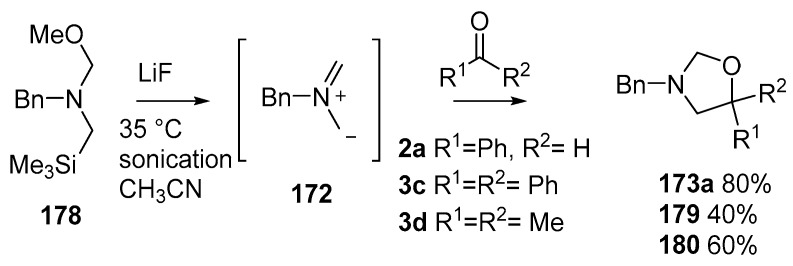
Cycloaddition of non-stabilized ylide **172**, derived from methoxymethylamine reagent **178**, with benzaldehyde (**2a**) and ketones **3c** and **3d**.

By far the most studied and utilized of the α-substituted methyl(trimethylsilylmethyl)amine reagents is the *N*-methoxymethyl-substituted reagent **178** [129,130,131], readily produced from benzylamine by alkylation with chloromethyltrimethylsilane followed by reaction with formaldehyde in the presence of methanol [132]. The reagent is now commercially available from various vendors. The advantages of this reagent include that the ylide **172** can be produced by treatment with LiF, without the use of Ag salts, by sonication at 35 °C in acetonitrile and when reacted with benzaldehyde (**2a**), cycloadduct **173a** was obtained in a higher yield than that obtained with the previous reagents. When **178** was reacted in this way with ketones, benzophenone (**3c**) or acetone (**3d**), moderate yields of the corresponding cycloadducts **179** or **180** were obtained (Scheme 44).

**Scheme 45:**
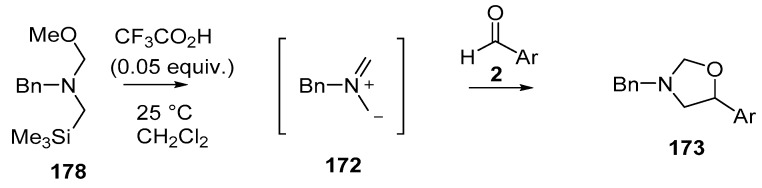
Cycloaddition of non-stabilized ylide **172**, generated by CF_3_CO_2_H-catalyzed decomposition of methoxymethylamine reagent **178**, with aromatic aldehydes **2**.

**Figure 2:**
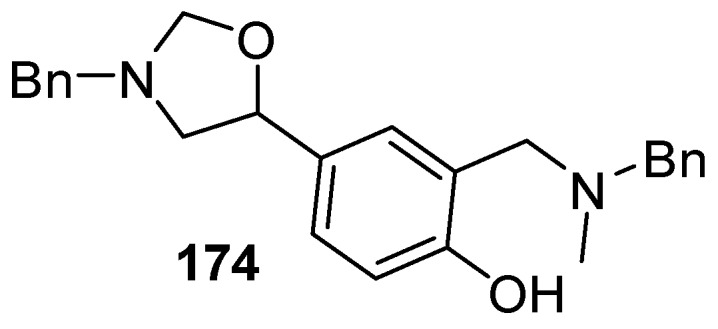
The *bis* adduct isolated from the reaction of 4-hydroxybenzaldehyde (**2t**, Table 23, entry 16).

Recently, a thorough exploration of the reactions of reagent **178** with aromatic and heteroaromatic aldehydes **2** was reported [133]. Firstly, it was found that generating the ylide by trifluoroacetic acid catalysis [131] in CH_2_Cl_2_ at 25 °C in the presence of benzaldehyde (**2a**) afforded a 95% yield of the oxazolidine cycloadduct **173a** (Scheme 45) (Table 23, entry 1), a higher yield than that reported using LiF to generate the ylide from reagent **178** [129]. The trifluoroacetic acid catalysis method was then applied to a range of aromatic aldehydes **2** (Table 23). High yields of cycloadducts **173** were obtained in the cases of benzaldehydes substituted with electron-withdrawing (entries 2–7), electron-donating (entries 8–11, 14), sterically demanding (entries 5 and 12), basic (entry 8) and acidic (entry 13) groups. Chemoselectivity was seen in the case of the 4-cyano example (entry 7) with only the product from cycloaddition to the aldehyde moiety being obtained. There were some limitations, in that 3-hydroxybenzaldehyde (**2s**) afforded complex mixtures and oxazolidine **173o** could not be detected. Additionally, for 4-hydroxybenzaldehyde (**2t**), the main product was the *bis* adduct **174**, isolated in 53% yield (Figure 2).

The cycloaddition of ylide **172**, generated by trifluoroacetic acid catalyzed decomposition of silylamine reagent **178**, to a range of heteroaromatic aldehydes **2** was also explored with the only limitation appearing to be electron-rich aromatic aldehydes (Table 24) [133]. 2-Furan-, 2-thiophene- and 3-pyridine carboxaldehydes (**2u**–**w**) all underwent efficient cycloaddition under these conditions (Entries 1–3). In contrast, experiments with pyrrole-2-carboxaldehyde (**2x**) and *N*-methylpyrrole-2-carboxaldehyde (**2y**) afforded complex intractable product mixtures with no sign of oxazolidine products (Entries 4 and 5). The lack of effective cycloaddition under these conditions was thought to be due to the electron-rich nature of the pyrrole leading to deactivation of the pyrrole via increasing the carbonyl LUMO energy with concomitant increase in the carbonyl LUMO-azomethine ylide HOMO energy gap. Consistent with this rationale, when the pyrrole contained the electron-withdrawing *N*-benzenesulfonyl group (compound **2z**), which was expected to lower the carbonyl LUMO energy, a high yield of the oxazolidine product **173v** was obtained.

**Figure 3:**
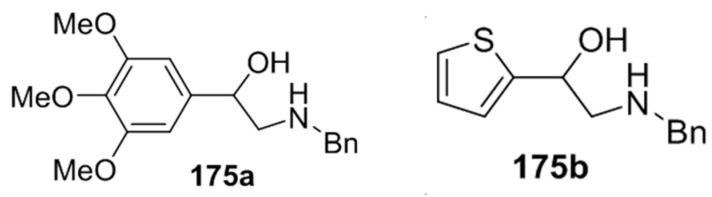
Products from hydrazinolysis of oxazolidines **173j** and **173r**.

Recently, 5-aryloxazolidines **173**, formed under the above conditions, were applied to the synthesis of β-hydroxy-β-arylethylamines [87]. The oxazolidines **173j** and **173r** underwent hydrazinolysis leading to the trimethoxyphenyl- and thiophenyl derivatives **175a** and **175b**, isolated in 68% and 71%, respectively (Figure 3).

**Scheme 46:**
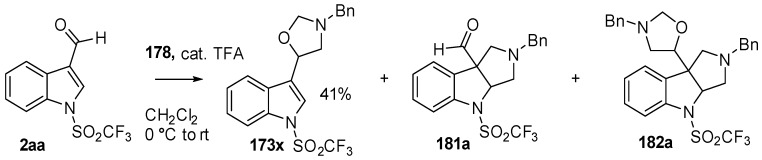
Cycloaddition of azomethine ylide **172**, formed from silylamine reagent **178**, with indole-3-carboxaldehyde **2aa**.

**Scheme 47:**
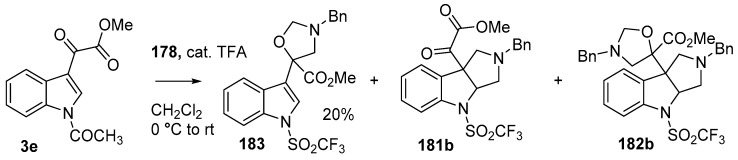
Cycloaddition of azomethine ylide **172**, formed from silylamine reagent **178**, with indole-3-pyruvate **3e**.

**Scheme 48:**
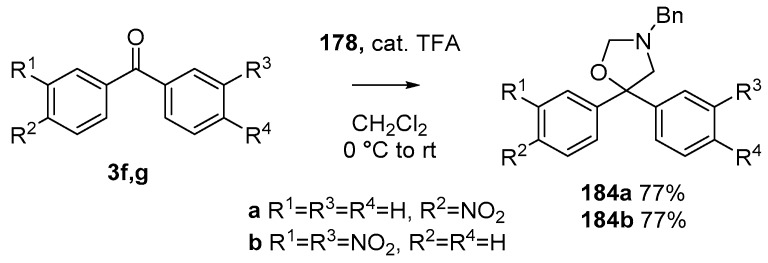
Cycloaddition of azomethine ylide **172**, formed from silylamine reagent **178**, with benzophenones **3f** and **3g**.

**Scheme 49:**
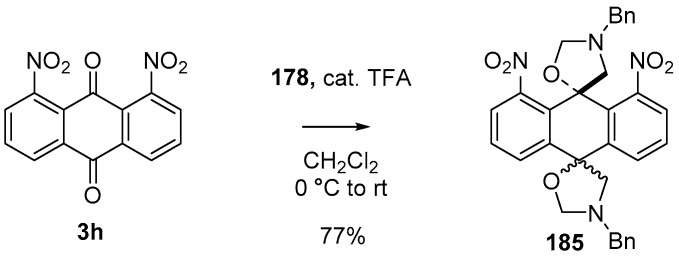
Cycloaddition of azomethine ylide **172**, formed from silylamine reagent **178**, with anthroquinone **3h**.

During a recent study into the dearomatization of electron-deficient arenes and heteroarenes by azomethine ylide cycloadditions, competing cycloadditions to aldehydes and ketones were occasionally observed [134]. The reaction of *N*-triflylindole-3-carboxaldehyde (**2aa**) with azomethine ylide **172**, formed by trifluoroacetic acid-catalyzed decomposition of reagent **178**, afforded a mixture of three products in a 6:2:2 ratio. The major product produced was the mono aldehyde adduct **173x** (isolated in 41% yield) with the balance of the product being an inseparable mixture of mono indole C2-C3 adduct **181a** and a bis-adduct **182a** formed by addition to both carbonyl and indole C2-C3 double bonds (Scheme 46). Similarly, methyl *N*-acetyl-indole-3-pyruvate (**3e**) afforded a similar distribution of a mono ketone adduct **183** (70% of crude product, 20% isolated yield) along with a mixture of C2-C3 double bond adduct **181b** and bis-adduct **182b** (~30% of crude product) (Scheme 47). The reaction of the same ylide with 4-nitro- and 3,3′-dinitrobenzophenones (**3f**) and (**3g**) resulted in exclusively ketone adducts **184a** and **184b** both isolated in 77% yield (Scheme 48). In a further example, 3,3′-dinitroanthroquinone (**3h**) afforded bis ketone adduct **185** isolated in 66% yield as a 9:1 mixture of diastereoisomers (Scheme 49). These studies indicate the relatively greater reactivity of such aldehyde and ketone groups over the nitroarene in these particular systems.

**Scheme 50:**
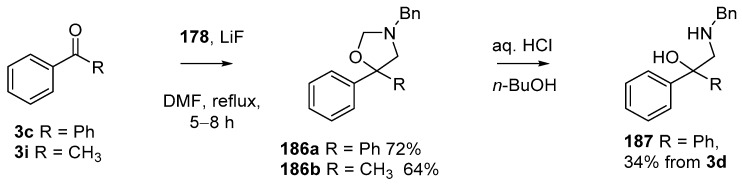
Cycloaddition of azomethine ylide **172**, formed from silylamine reagent **178**, with aromatic ketones **3c** and **3i**.

Using modified conditions (LiF, DMF, reflux), reagent **178** has been employed in azomethine ylide cycloadditions to benzophenone (**3c**) and acetophenone (**3i**), to produce good yields of the corresponding oxazolidines **186a** and **186b**, respectively (Scheme 50) [90]. Acid catalyzed hydrolysis of **186a** provided access to 2-amino-1,1-diphenylethylalcohol derivative **187**.

**Scheme 51:**
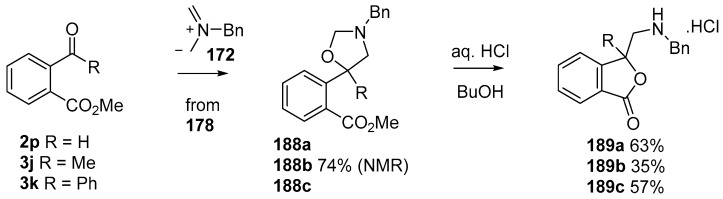
Cycloaddition of azomethine ylide **172**, formed from silylamine reagent **178**, with aromatic aldehyde **2p** and aromatic ketones **3j** and **3k**.

**Scheme 52:**
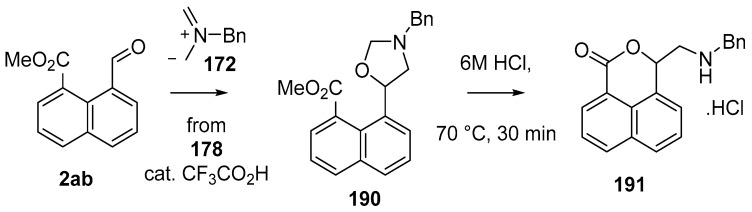
Cycloaddition of azomethine ylide **172**, formed from silylamine reagent **178**, with aromatic aldehyde **2ab**.

**Scheme 53:**
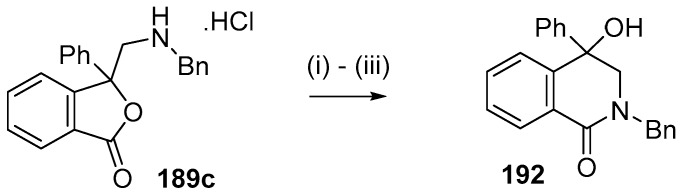
Lactamization of isobenzofuranone **189c**. *Reaction Conditions*: (i) NaHCO_3_; (ii) NaOH; then NH_4_Cl; (iii) *n*-BuOH, reflux.

**Scheme 54:**
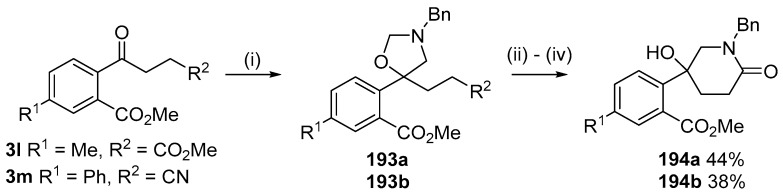
Cycloaddition of ylide **172** with aromatic ketones **3l** and **3m** followed by hydrolysis and condensation reactions to afford piperidones **194**. *Reaction Conditions*: (i) **178** (2.5 equiv.), LiF, DMF, reflux, 5 h; (ii) aq. HCl, *n*-BuOH, 90 °C; (iii) NH_4_Cl;(iv) *n*-BuOH, reflux.

This alkylaminomethylation chemistry was applied to aryl aldehyde and aryl ketone derivatives to produce alkylaminomethylated species that could undergo further transformations to produce alkylaminomethylphthalides (Scheme 51) [91]. Methyl 2-formyl benzoate (**2p**) underwent cycloaddition reaction with azomethine ylide **172** generated by trifluoroacetic acid catalyzed decomposition of reagent **178** to give a quantitative yield of oxazolidine **188a** which underwent hydrolysis and cyclization under acidic conditions at 70 °C to give phthalide **189a**. The less reactive dipolarophile methyl ester of 2-acetylbenzoic acid (**3j**) gave low yields of the oxazoldine **188b** under conditions involving trifluoroacetic acid catalysis; however, with the use of excess LiF in refluxing DMF a 74% yield of oxazoldine **188b** was obtained. Acidic hydrolysis and heating at 90 °C afforded phthalide **189b** in 35% yield from the ketone starting material. Similar treatment of the methyl ester of 2-benzoylbenzoic acid (**3k**) afforded phthalide **189c** in 57% yield. In the case of the methyl ester of 8-formyl-naphthalene-1-carboxylic acid (**2ab**), the alkylaminomethyl lactone **191** was obtained in 40% yield (Scheme 52). Neutralisation of phthalide **189c** followed by heating in refluxing butanol gave a 60% yield of the corresponding cyclized dihydroisoquinolinone **192** (Scheme 53). In related cycloaddition-hydrolysis-cyclization processes, the aryl ketone derivatives **3l** and **m** were elaborated into the respective 4-aryl-4-hydroxypiperidone derivatives **194a** and **b** (Scheme 54).

**Scheme 55:**
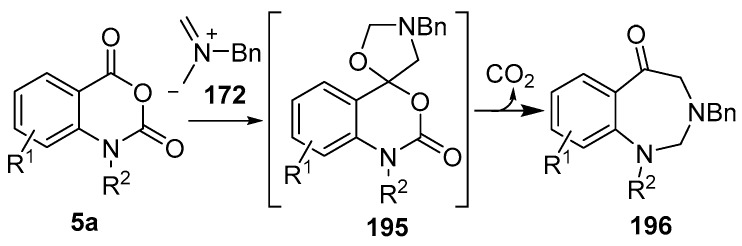
Cycloaddition of azomethine ylide **172**, formed from silylamine reagent **178**, with isatoic anhydrides **5a**, followed by conversion of the oxazolidines **195** to 1,3-benzodiazepin-5-ones **196**.

**Scheme 56:**
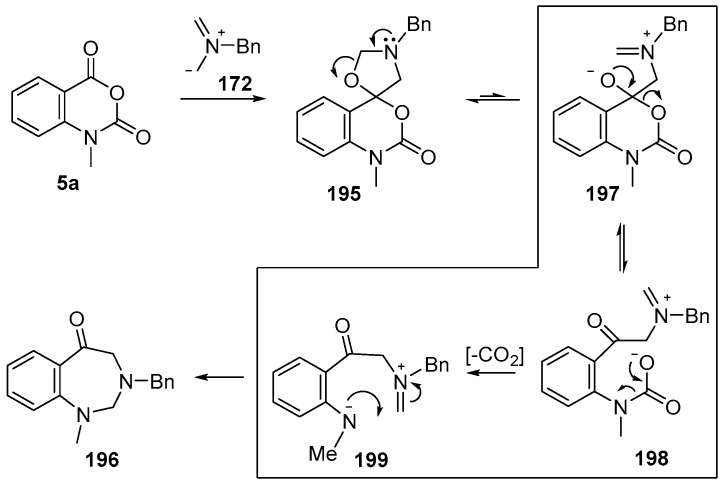
Proposed mechanism for the conversion of isatoic anhydrides **5a** into benzodiazepinones **196**.

Recently it was reported that activated carboxyl systems can undergo 1,3-dipolar cycloaddition reactions [40]. A range of isatoic anhydrides **5a** reacted with the azomethine ylide **172**, generated from reagent **178** by trifluoroacetic acid-mediated catalysis or by treatment with LiF, to afford spiro-oxazolidine cycloadducts **195**, that spontaneously extruded CO_2_ with structural rearrangement to give the isolated 1,3-benzodiazepin-5-one products **196** (Scheme 55). High yields of the benzodiazepinones **196** were obtained for a wide range of *N*- and benzo-substituted isatoic anhydrides except for cases with electron-releasing substituents ortho- or para-related to the dipolarophilic carboxyl group, in which case, starting material was returned (Table 25 and Table 26). The lack of reaction in these cases was thought to be due to stereoelectronic effects which resulted in raising the energy of the carbonyl LUMO energy and concomitant raising of the energy of the cycloaddition transition state. A plausible mechanism for the transformation of the isatoic anhydrides into the benzodiazepines was proposed involving a complex reaction cascade (Scheme 56). The initial cycloaddition gives the oxazolidine product (observable by NMR and IR spectroscopies) which undergoes step-wise oxazolidine ring-opening, decarboxylation and ring-closure.

In a recent development, phthalic anhydrides **5b** were reported to undergo cycloaddition with the azomethine ylide **172**, formed from reagent **178** under conditions of trifluoroacetic acid catalysis [41]. The generated spiro-oxazolidine cycloadducts **200** proved to be more stable than the analogous cycloadducts generated from isatoic anhydrides **5a**. Although the cycloadducts **200** decomposed during attempted purification on silica, they could be isolated in pure form after chromatography on Florosil™. The reaction appeared to be general with a range of substituted phthalic anhydrides reacting under these conditions and high yields of the cycloadduct products **200** were obtained (Table 27). In all cases only mono cycloaddition products were isolated. In the cases of the unsymmetrical phthalic anhydrides (Entries 4–6), inseparable mixtures of the two possible mono adducts were obtained, with varying degrees of regioselectivity. In the case, of 3-methoxyphthalic anhydride (Entry 6), a 70:30 ratio of the regioisomeric products were obtained, with the major product being that where the ylide adds to the carboxyl group distal to the methoxy group. This result was explained to be a combination of steric and electronic effects of the methoxy group on the adjacent carboxyl group having the effect of raising the transition state energy and lowering the rate of reactivity of the adjacent carboxyl group.

The reductive ring-opening of the spiro-oxazolidines **200** was also investigated [41]. Treatment of the oxazolidines with NaBH_4_ in methanol led to conversion, in reasonable yields, to 3-hydroxy-isobenzofuran-1-ones **201** (Table 28). The products were stable to chromatographic purification on silica which allowed for separation and characterization of the isomers (Entries 4 and 5). Additionally, the products appear to be mainly present as the depicted ring-closed form (rather than the tautomeric ring-opened keto-carboxylic acid form) in solution state, as assessed by NMR analyses and in solid state as shown by X-ray crystallographic analysis of a representative example. Lower yields obtained for two examples (Entries 7 and 8) were thought to be due to the electron-deficient nature of these systems resulting in over reduction side-reactions.

#### 2.5.3. Photochemistry of *N*-(silylmethyl)phthalimides and Related Reagents

**Scheme 57:**
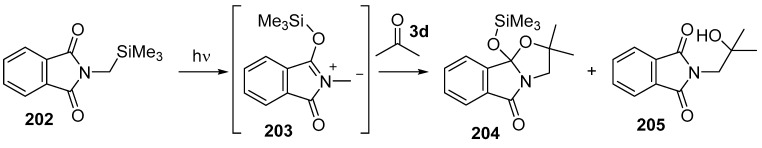
Cycloaddition of azomethine ylide **203**, generated by photochemical rearrangement of *N*-(silylmethyl)phthalimide (**202**), with acetone (**3d**).

An investigation of the photochemistry of *N*-trimethylsilylmethyl-substituted phthalimides revealed a C- to O-trimethylsilyl transfer process that produced azomethine ylide intermediates [135,136]. Irradiation of an acetonitrile solution of *N*-trimethylsilylmethylphthalimide (**202**) with Pyrex-filtered light led to migration of the silyl group from carbon to oxygen to form azomethine ylide **203** (Scheme 57). When performed in the presence of dipolarophiles, cycloadditions proceeded, and in the case where the reaction was performed in acetone (**3d**), a single cycloadduct **204** was obtained in 84% yield together with an alcohol side-product **205**. The alcohol side-product was presumed to be formed from hydrolysis of the cycloadduct in the reaction mixture and indeed the cycloadduct **204** could be transformed into the alcohol **205** by treatment with aqueous HCl.

#### 2.5.4. Electrochemical Oxidation of bis(silylmethyl)amines

**Scheme 58:**
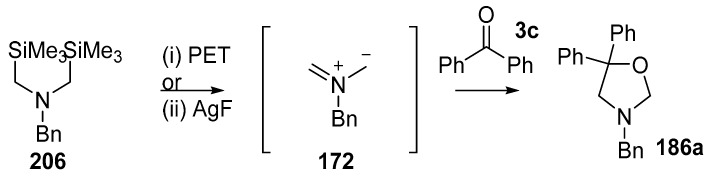
Cycloaddition of **172**, formed by electrochemical oxidation of **206**, with benzophenone **3c**. *Reaction conditions*: (i) **206**, 1,4-dicyanonaphthalene (0.20 equiv.), **3d** (1.6 equiv.), 80%; (ii) **206**, AgF (2 equiv.), **3d** (1.2 equiv.), CH_3_CN, 62%.

Double desilylation of bis(silylmethyl)amines using either photoelectron induced transfer (PET) or AgF were developed as processes for generating non-stabilised azomethine ylides [137]. In the case of bis(trimethylsilylmethyl)benzylamine (**206**) this process is an alternative method for generating ylide **172**. Whilst most of the examples involved the use of alkene dipolarophiles, benzophenone (**3c**) was also studied resulting in cycloadduct **186a** being isolated in 80% and 62% yields from PET and AgF processes, respectively (Scheme 58).

### 2.6. Reactions of Ylides Formed from Addition of Carbenes and Carbenoids to Schiff Bases

The reaction of carbenes or carbenoid species with imines is a convenient way to generate azomethine ylides and has been applied to cycloadditions with a range of carbonyl dipolarophiles.

#### 2.6.1. Diazoalkanes

**Scheme 59:**
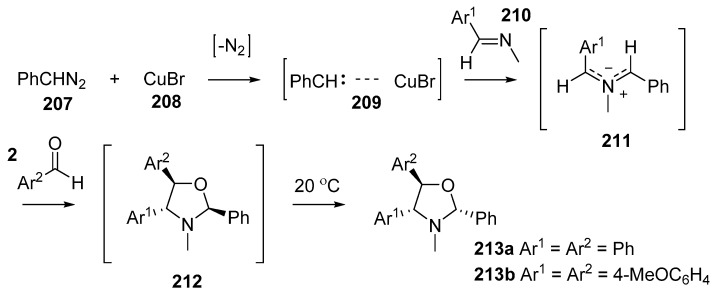
Cycloaddition of azomethines **211**, generated from CuBr-catalyzed decomposition of phenyldiazomethane (**207**) in the presence of imines **210**, with aromatic aldehydes **2**.

The catalytic decomposition of diazoalkanes in the presence of a Schiff’s base is a convenient source of azomethine ylides (Scheme 59) [138]. The copper(I) bromide-catalyzed decomposition of phenyldiazomethane **207** produces a carbene-Cu(I) complex **209**. In the presence of a large excess of imine **210** a *trans*-azomethine ylide **211** is formed and this intermediate reacts with dipolarophiles to product five-membered heterocycles. With benzaldehyde (**2a**) or anisaldehyde (**2c**) as the dipolarophile, oxazolidines **212** were formed, however they proved to be unstable and on standing at room temperature for a few days transformed into the more thermodynamically stable isomers **213**. Competition experiments were performed that indicated dimethyl maleate was a more reactive dipolarophile than benzaldehyde (**2a**).

**Scheme 60:**
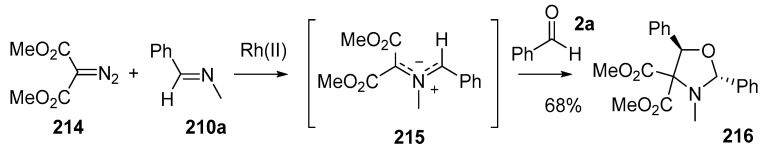
Cycloaddition of azomethine ylide **215**, generated from Rh(II)-catalyzed decomposition of diazomalonate **214** in the presence of imine **210a**, and benzaldehyde (**2c**).

The Rh(II)-catalyzed decomposition of α-diazocarbonyls in the presence of imino π-bonds is also an elegant way to produce functionalized azomethine ylides [139]. Many variations of this process were explored, including the use of benzaldehyde (**2a**) as a dipolarophile. The Rh(I)-catalyzed decomposition of diazomalonate **214** in the presence of imine **210a** provided stabilized azomethine ylide **215** which underwent cycloaddition to benzaldehyde (**2a**) to provide cycloadduct **216**, isolated as a single diastereoisomer in 68% yield (Scheme 60).

**Scheme 61:**
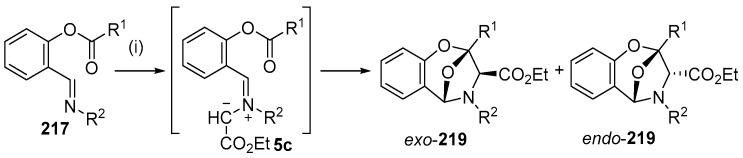
Intramolecular cycloaddition of azomethine ylide and ester carbonyl of intermediate **5c**. *Reagents and Conditions*: (i) N_2_CCHCO_2_Et **218**; Cu(tfacac)_2_, CH_2_Cl_2_, 40 °C.

**Scheme 62:**
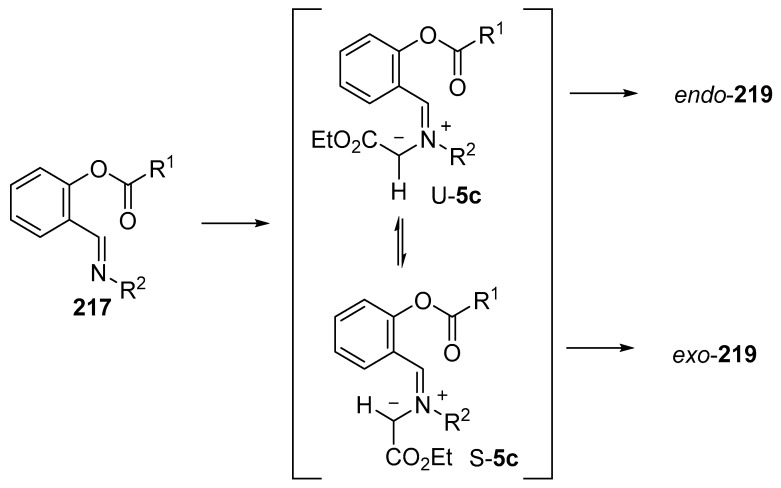
Mechanistic rationale for the formation of *endo*- and *exo*-cycloadducts 219.

As an extension to studies on the intramolecular addition of azomethine ylides derived from imines to ester carbonyl moieties (see Section 2.6.2), the intramolecular reaction of imines of *O*-acylsalicylic aldehydes with metallocarbenes generated from diazocarbonyl compounds was recently investigated experimentally and computationally [140]. A range of Rh- and Cu-catalysts were tested with copper(II)trifluoroacetoacetate (Cu(tfacac)_2_) providing the highest yields (Scheme 61). The reaction of imine **217** with ethyl diazoacetate (**218**) catalyzed by 10 mol % of Cu(tfacac)_2_, in refluxing benzene, led to formation of azomethine ylide intermediate **5c** which underwent intramolecular cycloaddition onto the ester carbonyl to produce both *endo* and *exo* isomers of **219**, isolated in 13% and 14% yield, respectively. In refluxing CH_2_Cl_2_, *endo*-**219** was obtained in 36% yield, whereas with the use of a stoichiometric amount of Cu(tfacac)_2_, a 41% yield of *endo*-**219** resulted. The latter conditions were applied to a wide range of imines (Table 29). In order for cycloaddition to occur, an electron-withdrawing substituent was required on the imine aryl group (Entries 1–8). In these cases, where an electron-withdrawing substituent was also present on the benzoyl group, both *endo*- and *exo*-cycloadduct isomers were produced (Entries 6–8). For the subset of cases with an electron-donating substituent on the benzoyl group, exclusive *endo* stereochemistry resulted (Entries 1–5). A range of experimental and theoretical (DFT) calculations were performed that indicated that the change in stereochemistry of cycloaddition was due to a decrease in the barrier to cycloaddition and an increase in the barrier of U- to S-ylide interconversion with an electron-withdrawing group on the benzoyl group. The U-ylide leads to the *endo*-cycloadduct, whereas the S-ylide leads to the *exo*-cycloadduct (Scheme 62).

#### 2.6.2. Reaction with Ylides Formed by Interaction of Imines with Dihalocarbenes

**Scheme 63:**
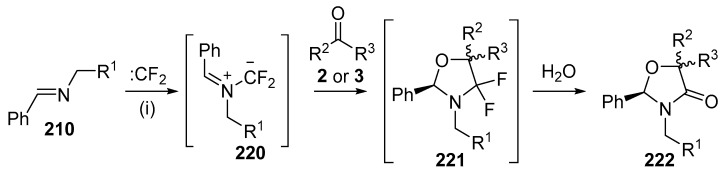
Cycloaddition of azomethine ylides **220** with aldehydes **2** or ketones **3**. *Reaction conditions*: (i) imine **210**, CBr_2_CF_2_, Pb powder, TBAF, carbonyl compound, CH_2_Cl_2_.

Azomethine ylides derived from the reaction of imines with difluoro- or dichlorocarbene also undergo cycloaddition with a range of carbonyl dipolarophiles. The first report of these processes demonstrated that ylides derived from difluorocarbene undergo regioselective cycloaddition with aldehydes and ketones to give oxazolidinone derivatives (Scheme 63, Table 30) [141]. Heating a mixture of the imine **210** (R^1^ = Ph), dibromodifluoromethane, Pb powder, tetrabutylammonium bromide and a twofold excess of benzaldehyde (**2a**) gave the oxazolidinone **222a** in 39% as a ca. 5:3 mixture of diastereoisomers (Entry 1). Presumably, the oxazolidinone **222a** was formed by hydrolysis of the cycloadduct **221a** during silica chromatography. The reaction with other aldehydes provided moderate yields of oxazolidinones **222** (Entries 2–5), however, the experiments with acetone (**3d**) or acetophenone (**3i**) gave low or no yield of product (Entries 6 and 7).

**Scheme 64:**
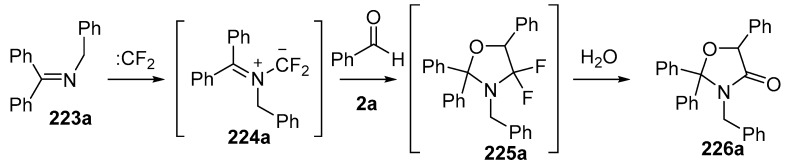
Cycloaddition of azomethine ylide **224a**, generated from difluorocarbene and imine **223a**, with benzaldehyde (**2a**).

**Scheme 65:**
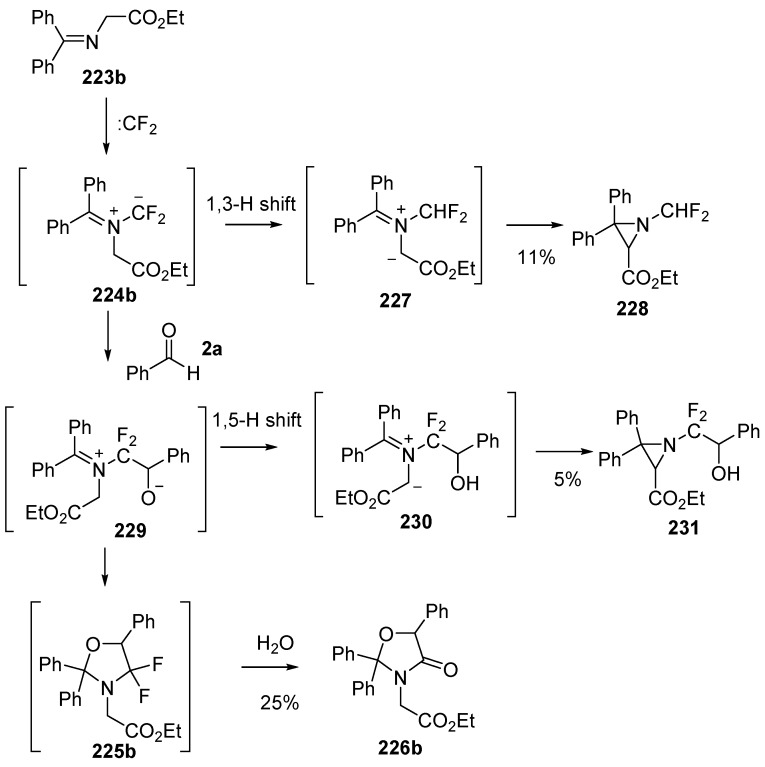
Cycloaddition of azomethine ylide **224b**, generated from difluorocarbene and imine **223b**, with benzaldehyde (**2a**).

Dipolar cycloaddition reactions starting with benzophenone imines **223** and difluorocarbene were also studied [141]. Under the same conditions as above, imine **223a** added to difluorocarbene to generate the azomethine ylide intermediate **224a**, which underwent efficient cycloaddition to benzaldehyde **(2a)** to afford the cycloadduct **225a**. The oxazolidine **225a** hydrolysed on silica to give the oxazolidinone **226a** which was isolated in 75% yield (Scheme 64). The reaction with the *N*-carboxymethyl ester **223b** under these conditions with benzaldehyde (**2a**) as the dipolarophile resulted in formation of oxazolidinone **226b** via ylide cycloaddition and hydrolysis along with aziridine side-products **228** and **231** (Scheme 65). The mechanism proposed for the formation of **228** involved a 1,3-H shift of the ylide intermediate **224a** to give an alternative ylide **227** which ring-closed to afford the isolated aziridine **228**. Alternatively, addition of benzaldehyde (**2a**) to the ylide **224b** could give ring-opened adduct **229** which could undergo a rapid 1,5 H-shift to give another alternative ylide **230** which on ring closure would afford aziridine **231**. The authors propose that ring-opened adduct **229** could ring-close to give oxazolidine **225b** which when exposed to silica, underwent hydrolysis to afford the isolated oxazolidinone **226b**. An alternative pathway whereby **224b** undergoes 1,3-dipolar cycloaddition to afford oxazolidine **225b** which then ring-opens to provide **229** eventually leading to **231**, was not mentioned.

**Scheme 66:**
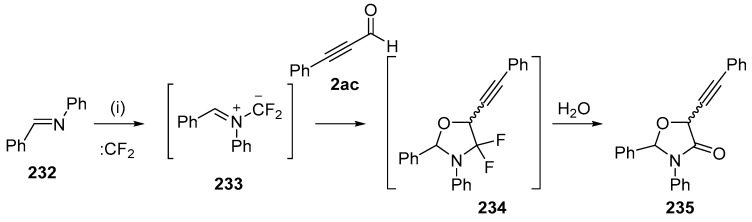
Regioselective cycloaddition of azomethine ylide **233** to the aldehyde of **2ac**. *Reaction conditions*: (i) imine **232**, CBr_2_CF_2_, Pb powder, TBAF, **2ac**, CH_2_Cl_2_.

A later study of addition of the ylides formed by imines and difluorocarbene with alkynes revealed side reactions involving addition to alkynyl aldehydes [142]. The ylide **233**, formed from *N*-phenylbenzaldehyde imine (**232**) and difluorocarbene, reacted with alkynyl aldehyde **2ac** to form the cycloadduct **234**. On silica chromatography, hydrolysis occurred and a mixture of oxazolidinones **235** was isolated in 25% yield as a 5:1 mixture of diastereoisomers (Scheme 66). In contrast, the corresponding alkynyl esters and alkynyl ketones resulted in selective addition to the alkyne triple bond.

**Scheme 67:**
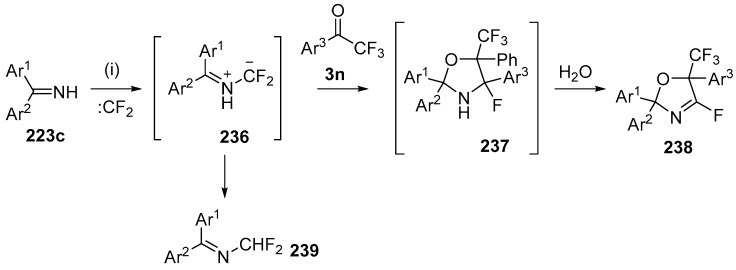
Cycloaddition of azomethine ylides **236** to trifluoroacetophenones **3n**. *Reagants and Conditions*: (i): imine **223c**, Pb (3 equiv.), Bu_4_NBr (3 equiv.), CF_2_Br_2_ (3 equiv.), Ar^1^COCF_3_ **3** (3 equiv.), CH_2_Cl_2_, ultrasound.

The cycloaddition of *gem*-difluorosubstituted *NH*-azomethine ylides with α,α,α-trifluoro-acetophenones has been reported to form 4-fluorooxazolidines [143]. The optimized procedure involved stirring a mixture of the imine **223c**, with an excess of trifluoroacetophenone (**3o**), CBr_2_F_2_, Pb filings and tetrabutylammonium bromide, and resulted in isolation of the fluoro-oxazolines **238** after chromatography on silica (Scheme 67, Table 31). Presumably the imine **223c** reacts with liberated difluorocarbene to afford the azomethine ylide **236** which undergoes cycloaddition with trifluoroacetophenones **3** to afford the cycloadducts **237**. On exposure to silica, the cycloadducts **237** lose HF to afford the 2-fluorooxazolidine products **238**. A side-product **239** was at times isolated, that was thought to be formed by the isomerization of the ylide **236**. The optimized method was applicable to a range of substituted benzophenone imines **223c** and substituted trifluoroacetophenones **3** (Table 31). Additionally the products appear to have potential utility, with **238** undergoing displacement of the fluoride with a range of *N*- and *O*-centred nucleophiles.

**Scheme 68:**
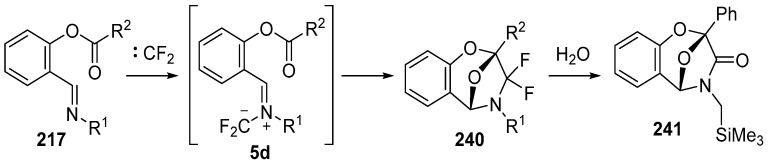
Intramolecular cycloaddition of the azomethine ylide and ester moieties of intermediate **5d**.

**Scheme 69:**
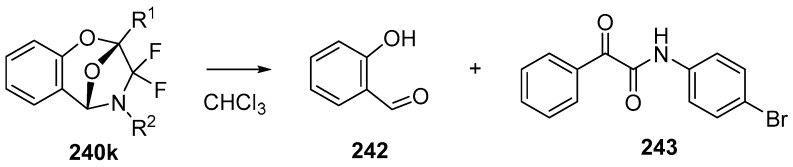
Decomposition of cycloadduct **240k** in chloroform.

**Scheme 70:**
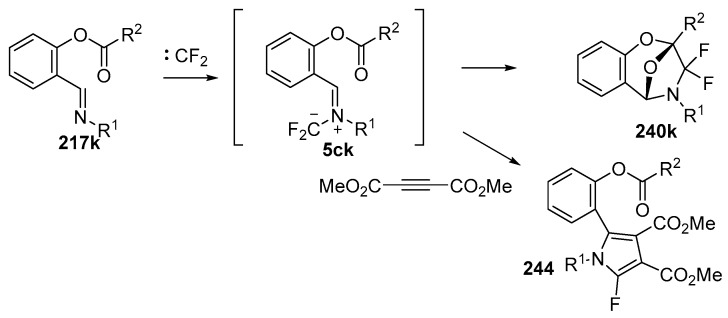
Intramolecular versus intermolecule competition reactions of azomethine ylides **5ck**.

**Figure 4:**
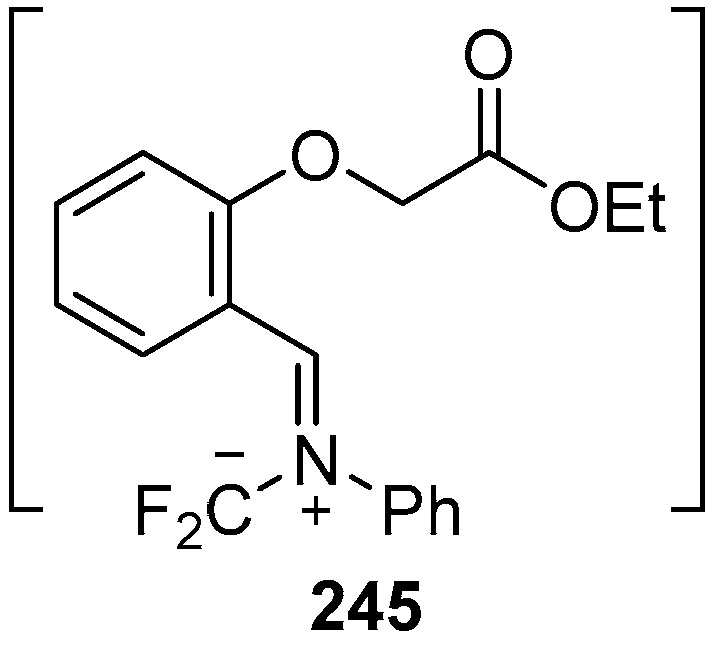
Analogous azomethine ylide that fails to undergo intramolecular cycloaddition.

In 2002, the first 1,3-dipolar cycloaddition of an azomethine ylide to an ester carbonyl was reported [144,145]. Azomethine ylides **5d**, generated by the reaction of difluorocarbene with aryl and alkyl imines of *O*-acylated salicylaldehydes **217**, underwent intramolecular 1,3-dipolar cycloaddition onto the ester carbonyl to give cycloadducts **240** (Scheme 68). This process proved to be quite general for a range of *N*-aryl substituted imines as well as *N*-alkylimines (Table 32). The cycloadducts **240a**–**m** were stable enough to be isolated and characterized whereas the cycloadduct **240n**, from the *N*-(trimethylsilylmethyl) imine, underwent rapid hydrolysis during chromatographic purification on silica, affording lactam **241** in 67% yield. Interestingly, the cycloadducts **240** degraded on longer term storage in chloroform, yielding, for example, from **240k** after 3.5 months, salicylaldehyde (**242**) and phenylglyoxamide **243** (Scheme 69). In the cases of imines **217f**–**i** there is an internal competition between cycloaddition of the ylide to the ester carbonyl and cycloaddition to the olefin. Only cycloaddition to the ester carbonyl was observed indicating a much faster rate of cycloaddition to the ester than these types of olefins. An external competition reaction was performed by running the reaction of **217k** in the presence of a reactive dipolarophile, dimethyl acetylenedicarboxylate, and resulted in roughly equimolar amounts of intramolecular cycloadduct **240k** and the pyrrole **244** resulting from intermolecular cycloaddition of the ylide with the alkyne (Scheme 70). Additionally the ylide **245** (Figure 4) did not undergo intramolecular cycloaddition, indicating the requirement of the close proximity of the ester group to the ylide for efficient ester carbonyl cycloaddition to occur.

**Scheme 71:**
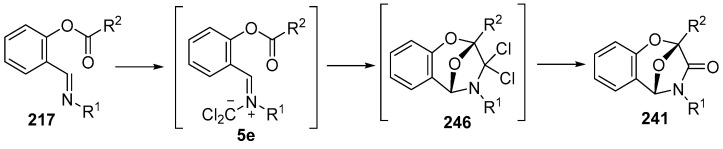
Intramolecular cycloadditions of the azomethine ylide **5e**.

The reaction of analogous ylides derived from imines and dichlorocarbene has also been explored [146]. Although *N*-benzylidene anilines generally react with dichlorocarbene to give aziridines, the salicylaldehyde imines **217** reacted with dichlorocarbene, generated under a number of different conditions, to produce 2,5-epoxy-1,4-benzoxepin-3-ones **241**, isolated in high yields (Scheme 71, Table 33). The proposed mechanism involves addition of the dichlorocarbene to the imine **217** to produce **5e** which undergoes intramolecular cycloaddition of the ylide onto the ester carbonyl to give adduct **246** which then hydrolyses on work up or chromatographic purification, providing the isolated lactams **241**.

**Scheme 72:**
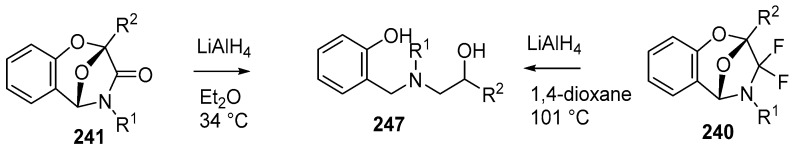
Reduction of cycloadducts **240** and **241**.

The chemistry of the bicyclic systems was also briefly explored. Reduction of **241b**, **o** or **i** with LiAlH_4_ in refluxing ether afforded the respective aminoalcohols **247b**, **o** or **i** (Scheme 72). The analogous reduction of the difluorinated cycloadducts **240a** and **m**, required more forcing conditions and was achieved in refluxing dioxane (Scheme 72, Table 34).

### 2.7. Miscellaneous Reactions

#### 2.7.1. Reactions with Ylides Formed by Reaction of Isocyanides, Alkylboranes and Aldehydes

**Scheme 73:**
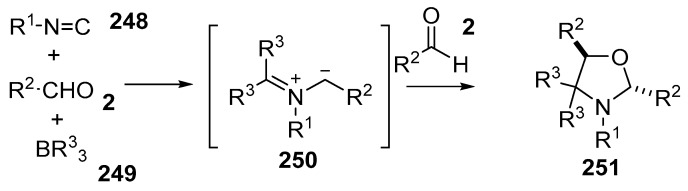
Cycloaddition of azomethine ylides, generated by the reaction of aldehydes **2**, isocyanides **248** and organoboron compounds **249**.

A multicomponent reaction of aldehydes **2**, isocyanides **248** and trialkylboron reagents **249** resulted in formation of azomethine ylides **250** which underwent further reaction with aldehydes **2** to yield oxazolidines **251** in an efficient manner, with the aldehyde group playing dual roles in the formation of the ylide as well as the dipolarophile (Scheme 73) [147]. The reaction generally worked for aryl isocyanides and aryl carboxaldehydes, however failed in the case of alkyl and alkenyl isocyanides and sterically hindered aldehydes (Table 35).

**Scheme 74:**
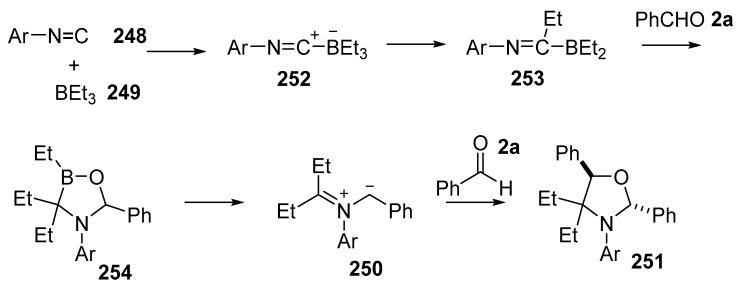
Proposed mechanism for the formation of oxazolidines **251**.

The mechanism that was proposed for this transformation involves a complex series of steps (Scheme 74). The process is initiated by addition of the isocyanide **248** to the trialkylborane **249** to give adduct **252**. Alkyl migration would then give iminoborane **253**. Further interaction of **253** with benzaldehyde **2a** with concomitant alkyl migration gives an oxazaborolidine intermediate **254**. Electrocyclic ring-opening of **254** affords the non-stabilized azomethine ylide **250**. Dipolar cycloaddition with further aldehyde **2a** would then provide for the *trans*-oxazolidine **251**.

#### 2.7.2. Reactions of Dicyanoazomethine Ylides

**Scheme 75:**
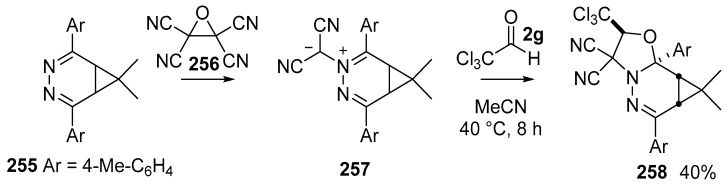
Cycloaddition of azomethine ylides **257** with trichloroacetaldehyde **2g**.

The stable dicyanoazomethine ylides **257**, derived from reaction of 3,4-diazanorcaradienes **255** with tetracyanoethylene oxide **256** [148], undergo reaction with a range of dipolarophiles [149]. The carbonyl dipolarophile trichloroacetaldehyde (**2g**) undergoes efficient cycloaddition with ylide **257** to give cycloadduct **258** isolated as a single *endo* stereoisomer in 40% yield (Scheme 75).

## 3. Conclusions

The 1,3-dipolar cycloaddition reaction of azomethine ylides and carbonyl dipolarophiles is a general method for producing oxazolidines. In terms of scope, many ylides have been demonstrated to react with carbonyl groups including an array of stabilized and non-stabilized ylides and many more ylide systems could be explored. Aldehydes including formaldehyde are the most studied group of carbonyl dipolarophiles studied, with ketones are also being well represented. There are few examples of ketenes, even though these systems appear quite reactive. Activated carboxyl systems such as isataoic anhydrides and phthalic anhydrides have been shown to react with non-stabilized azomethine ylides and the interesting reactivity of the spiro-fused oxazolidine products deserves further exploration. An intramolecular cycloaddition of azomethine ylides and esters (the latter group is typically unreactive in dipolar cycloadditions) provides for bicyclic systems and also warrants further exploration.

## Figures and Tables

**Table 1:** Cycloaddition of azomethine ylide **19** to aldehydes **2a**–**f** (Scheme 3).

Entry	R^1^	Product 20 (% Yield)
1	Ph	**20a** (100)
2	CH_3_	**20b** (60)
3	4-OMe-C_6_H_4_	**20c** (100)
4	PhCH=CH	**20d** (100)
5	*i*-Pr	**20e** (45)
6	Ph_2_CH	**20f** (80)

**Table 2:** Cycloaddition of *trans*-azomethine ylide **24** to aldehydes **2** (Scheme 4).

Entry	R^1^	R^2^	R^3^	R^4^	Oxazolidines 25 and 26 (4,5-*trans*:*cis* ^1^, % Yield)
1	3-NO_2_-C_6_H_4_	*i*-Pr	Ph	4-Cl-3-NO_2_-C_6_H_3_	**a** (61:39, 56)
2	4-NO_2_-C_6_H_4_	C_6_H_11_	Ph	4-Cl-3-NO_2_-C_6_H_3_	**b** (75:25, 44)
3	Ph	*i*-Pr	Ph	4-NO_2_-C_6_H_4_	**c** (100:0, 21)
4	3-NO_2_-C_6_H_4_	*i*-Pr	Ph	4-NO_2_-C_6_H_4_	**d** (45:55, 71)
5	4-NO_2_-C_6_H_4_	C_6_H_11_	Ph	4-NO_2_-C_6_H_4_	**e** (100:0, 25)
6	3-NO_2_-C_6_H_4_	*i*-Pr	Ph	2,4-(NO_2_)_2_-C_6_H_3_	**f** (0:100, 65)
7	3-NO_2_-C_6_H_4_	*i*-Pr	Ph	Ph	**g** (36:64, 48)
8	Ph	C_6_H_11_	Ph	4-NO_2_-C_6_H_4_	**h** (100:0, 45)
9	3-NO_2_-C_6_H_4_	C_6_H_11_	Ph	Ph	**i** (42:58, 44)
10	3-NO_2_-C_6_H_4_	C_6_H_11_	Ph	4-NO_2_-C_6_H_4_	**j** (32:68, 57)
11	Ph	*i*-Pr	Ph	CCl_3_	**k** (100:0, 84)
12	3-NO_2_-C_6_H_4_	*i*-Pr	Ph	CCl_3_	**l** (100:0, 65)
13	4-NO_2_-C_6_H_4_	C_6_H_11_	Ph	CCl_3_	**m** (100:0, 78)
14	Ph	Bn	4-MeC_6_H_4_	CCl_3_	**n** (100:0, 38)
15	3-NO_2_-C_6_H_4_	C_6_H_11_	Ph	CCl_3_	**o** (100:0, 72)

^1^ Determined by integration of ^1^H-NMR spectra.

**Table 3:** Cycloaddition of the azomethine ylides derived from aziridines **23** to diphenylcyclopropenone (**3a**) (Scheme 5).

Entry	R^1^	R^2^	R^3^	Product 29 (% Yield)
1	Ph	Me	Ph	**a** (20) ^1^
2	Ph	*i*-Pr	Ph	**b** (24) ^1^
3	Ph	*i*-Pr	4-NO_2_-C_6_H_4_	**c** (24) ^1^
4	Ph	C_6_H_11_	Ph	**d** (65)
5	4-NO_2_-C_6_H_4_	C_6_H_11_	Ph	**e** (23) ^1^
6	3-NO_2_-C_6_H_4_	C_6_H_11_	Ph	**f** (27) ^1^
7	4-NO_2_-C_6_H_4_	C_6_H_11_	Ph	**g** (64)
8	4-Ph-C_6_H_4_	C_6_H_11_	Ph	**h** (100)
9	Ph	C_6_H_11_	4-OMe-C_6_H_4_	**i** (79)
10	Ph	C_6_H_11_	4-Me-C_6_H_4_	**j** (20) ^1^
11	Ph	C_6_H_11_	4-NO_2_-C_6_H_4_	**k** (23)
12	Ph	C_6_H_11_	CH_3_	**l** (84)
13	4-Ph-C_6_H_4_	C_6_H_11_	CH_3_	**m** (59)

^1^ Used 1:1 aziridine to diphenylcyclopropenone; otherwise 4:3 aziridine to diphenylcyclopropenone.

**Table 4:** Cycloaddition of azomethine ylide **37** to aromatic aldehydes **2a** and **2i** (Scheme 7).

Entry	R^1^ = R^2^	R^3^	R^4^	Product 38 (% Yield)
1	H	4-NO_2_-C_6_H_4_	4-NO_2_-C_6_H_4_	**a** (80)
2	H	Ph	Ph	**b** (29)
3	H	4-NO_2_-C_6_H_4_	Ph	**c** (59)
4	Me	4-NO_2_-C_6_H_4_	4-NO_2_-C_6_H_4_	**d** (39)

**Table 5:** Cycloaddition of azomethine ylides **45** to aldehydes **2** catalyzed by nickel(II) perchlorate (Scheme 9).

Entry	Ar	R^1^	R^2^	% Yield 46
1	Ph	Me	Ph	67
2	Ph	Me	3-Me-C_6_H_4_	64
3	Ph	Me	4-*i*-Pr-C_6_H_4_	72
4	Ph	Me	4-OMe-C_6_H_4_	92
5	Ph	Me	2-OMe-C_6_H_4_	55
6	Ph	Me	4-Cl-C_6_H_4_	50
7	Ph	Me	4-Br-C_6_H_4_	55
8	Ph	Me	2-furyl	80
9	Ph	Me	1-napthyl	70
10	Ph	Me	(*E*)-Ph-CH=CH	87
11	Ph	Me	3,4,5-(OMe)_3_-C_6_H_2_	89
12	4-NO_2_-C_6_H_4_	Me	3,4,5-(OMe)_3_-C_6_H_2_	99
13	4-Cl-C_6_H_4_	Me	3,4,5-(OMe)_3_-C_6_H_2_	83
14	4-Br-C_6_H_4_	Me	3,4,5-(OMe)_3_-C_6_H_2_	97
15	4-Me-C_6_H_4_	Me	3,4,5-(OMe)_3_-C_6_H_2_	84
16	4-*i*-Pr-C_6_H_4_	Me	3,4,5-(OMe)_3_-C_6_H_2_	84
17	3-Me-C_6_H_4_	Me	3,4,5-(OMe)_3_-C_6_H_2_	88
18	2-Br-C_6_H_4_	Me	3,4,5-(OMe)_3_-C_6_H_2_	90
19	Ph	Et	3,4,5-(OMe)_3_-C_6_H_2_	82
20	Ph	*i*-Pr	3,4,5-(OMe)_3_-C_6_H_2_	90
21	4-*i*-Pr-C_6_H_4_	*i*-Pr	3,4,5-(OMe)_3_-C_6_H_2_	90
22	4-NO_2_-C_6_H_4_	Me	Ph	84
23	4-NO_2_-C_6_H_4_	Me	4-OMe-C_6_H_4_	96
24	4-NO_2_-C_6_H_4_	Me	4-Cl-C_6_H_4_	70
25	Ph	Me	3,4,5-(OMe)_3_-C_6_H_2_	82

**Table 6:** Zinc triflate-catalyzed cycloaddition of azomethine ylide **45** to aldehydes **2** (Scheme 9).

Entry	Ar	R^1^	R^2^	% Yield 46
1	Ph	Me	Ph	65
2	Ph	Me	2-Br-C_6_H_4_	51 ^1^
3	Ph	Me	4-Br-C_6_H_4_	62
4	Ph	Me	2-OMe-C_6_H_4_	81
5	Ph	Me	3-OMe-C_6_H_4_	77
6	Ph	Me	4-OMe-C_6_H_4_	84
7	Ph	Me	4-Me-C_6_H_4_	85
8	Ph	Me	4-NO_2_-C_6_H_4_	52
9	2-Br-C_6_H_4_	Me	4-Me-C_6_H_4_	87
10	2-Br-C_6_H_4_	Me	4-OMe-C_6_H_4_	74
11	3-Br-C_6_H_4_	Me	4-Me-C_6_H_4_	97
12	3-Br-C_6_H_4_	Me	4-OMe-C_6_H_4_	97
13	3-Br-C_6_H_4_	Me	4-Br-C_6_H_4_	94
14	4-Me-C_6_H_4_	Me	4-OMe-C_6_H_4_	84
15	Ph	Et	Ph	69
16	Ph	Et	4-Me-C_6_H_4_	74
17	Ph	Et	4-OMe-C_6_H_4_	72
18	Ph	Me	1-napthyl	73
19	Ph	Me	(*E*)-Ph-C=CH	78
20	3-Br-C_6_H_4_	Me	2-furyl	63

^1^ *cis*/*trans* = 1:1.

**Table 7:** Cycloaddition of azomethine ylide **51** to aromatic aldehydes **2** (Scheme 12).

Entry	R^1^	2,3-*trans*:*cis* ^1^	52/53 (% yield)
1	Ph	100:0 ^2^	**52a** (63%) ^2^
2	4-NO_2_-C_6_H_4_	66:34	**52b** (60%); **53b** (30%)
3	4-OMeC_6_H_4_	100:0	**52c** (37%)
4	4-pyridyl	80:20	**52d** (35%) ^3^
5	PhCO	100:0	**52e** (85%)

^1^ Ratio determined by ^1^H-NMR analysis; ^2^ traces of **53a** isolated; ^3^ **53d** not isolated.

**Table 8:** Reaction between glycine derivatives **47** or **54** and aromatic aldehydes **2a** or **2i** (Scheme 13).

Entry	R^1^	R^2^	Product Ratio ^1^	Product 55 (% Yield)
1	Me	4-NO_2_-C_6_H_4_	**55a** (48):**55b** (19):**56a** (17):**56b** (17)	**55a** (35%), **55b** (19%) ^2^
2	Me	Ph	**56a** (33):**56b** (24)	^3^
3	Bn	4-NO_2_-C_6_H_4_	**55a** (38):**55b** (23):**56a** (19):**56b** (21)	**55a** (35%), **55b** (19%) ^4^
4	Bn	Ph	^5^	**55a** (6%)

^1^ Composition determined by ^1^H-NMR analysis; ^2^ **56a**,**b** not isolated; ^3^ **56a**,**b** not stable to SiO_2_; ^4^ **56a**,**b** isolated as a mixture in 4% yield; ^5^ Not determined.

**Table 9:** Domino decarboxylative condensation, [3 + 2] cycloaddition and ring-opening aromatization process to give *N*-β-hydroxyethyl pyrroles **70** (Scheme 16).

Entry	R^1^	Product 70 % Yield (*dr*) ^1^
1	3-NO_2_-C_6_H_4_	95 (70:30)
2	2-NO_2_-C_6_H_4_	73 (53:47)
3	4-NO_2_-C_6_H_4_	85 (65:35)
4	3-CN-C_6_H_4_	68 (65:35)
5	4-CN-C_6_H_4_	75 (65:35)
6	4-CF_3_-C_6_H_4_	45 (60:40)
7	3-NO_2_-4-Cl-C_6_H_3_	83 (67:33)
8	3-NO_2_-4-OMe-C_6_H_3_	85 (73:27)
9	2-Cl-5-NO_2_-C_6_H_3_	87 (73:27)
10	3-CN-5-F-C_6_H_4_	72 (72:28)
11	3-pyridyl	82 (57:43)
12	CO_2_Et	43 (63:37)

^1^ Determined by ^1^H-NMR.

**Table 10:** Cycloaddition of azomethine ylide **79** to aldehydes **2** or ketones **3** (Scheme 18).

Entry	R^2^	R^3^	Oxapenams 80 % Yield (*exo*:*endo*) ^1^
1	Ph	H	19 (1:1)
2	4-NO_2_-C_6_H_4_	H	21 (1:1)
3	4-Cl-C_6_H_4_	H	26 (1:1)
4	4-CF_3_-C_6_H_4_	H	21 (1:1)
5	C_6_F_5_	H	40 (1:1.5)
6	CF_3_	CF_3_	56
7	fluorenyl		25

^1^ Assigned by NOE difference spectroscopy.

**Table 11:** Cycloaddition of azomethine ylide **81** to aldehydes **2** (Scheme 19).

Entry	Ar	Oxazolidines 82 % Yield
1	2-NO_2_-C_6_H_4_	95
2	3-NO_2_-C_6_H_4_	98
3	4-NO_2_-C_6_H_4_	92
4	4-Cl-C_6_H_4_	87
5	2,4-(Cl)_2_-C_6_H_3_	91
6	2-Br-C_6_H_4_	86
7	3,4-(OCH_2_O)-C_6_H_3_	58
8	1-naphthyl	72
9	2-pyridyl	77
10	2-thienyl	87

**Table 12:** Cycloaddition of azomethine ylide **81** to ketones **3** (Scheme 21).

Entry	Ketone 3	Oxazolidines 94 % Yield ^1^
1	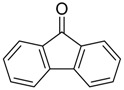	96 (61) ^2^
2	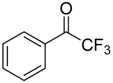	quant.
3	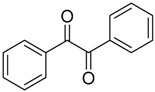	quant.
4	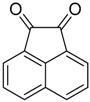	quant.
5	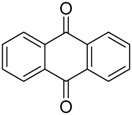	96
6	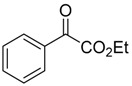	94
7	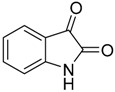	quant.
8	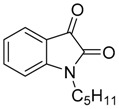	quant.
9	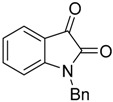	nd

^1^ Based on the ^1^H-NMR of the crude reaction mixture; ^2^ Isolated yield. nd = not determined.

**Table 13:** Cycloaddition of excess azomethine ylide **81** to 3-cyanochromones **96** (Scheme 22).

Entry	R	Oxazolidines 97 % Yield ^1^
1	H	quant.
2	Me	quant.
3	Cl	quant. (32) ^2^

^1^ Crude yield; ^2^ Yield after recrystallization.

**Table 14:** Ring-contraction of benz-1,2,6-oxadiazepines **99** to indazole-*N*-oxides **100** and formation of 3-methyl-5-aryloxazolidines **82b**–**e** (Scheme 23).

Entry	R^1^	R^2^	Indazole-*N*-oxides 100 % Yield	Oxazolidines 82 % Yield
1	H	H	40	43
2	~OCH_2_O~		32	30
3	OMe	OMe	34	38
4	Br	H	37	33

**Table 15:** Cycloaddition of *N*-metalated azomethine ylide **118** to benzaldehyde (**2a**) (Scheme 29) ^1^.

Entry	R^1^	R^2^	Oxazolidines 119 % Conversion ^2^ (*trans*:*cis*) ^3^
1	Ph	Me	56 (1.6:1)
2	4-F-C_6_H_4_	Et	63 (1.7:1)
3	4-F-C_6_H_4_	Et	82 (1.7:1)
4	4-CN-C_6_H_4_	Et	90 (2.3:1)
5	4-CF_3_-C_6_H_4_	Et	96 (2.3:1)
6	4-CN-C_6_H_4_	Et	80 (1.8:1) ^4^
7	4-CF_3_-C_6_H_4_	Et	93 (2.0:1) ^5^
8	4-CF_3_-C_6_H_4_	Me	84 (2.5:1)
9	4-CF_3_-C_6_H_4_	Me	78 (2.0:1) ^6^

^1^ Catalyst loading: AgOAc (10 mol %), PPh_3_ (20 mol %); solvent: THF. In entries 1 and 2, 5 mol % AgOAc and 10 mol % PPh_3_ used; ^2^ Determined by ^1^H- NMR; ^3^ The 4,5-*trans* to 4,5-*cis* ratio of oxazolidines **119** was determined by hydrolysis to the corresponding amino alcohols, and analysis of the ^1^H-NMR spectrum; ^4^ Reaction run at −20 °C; ^5^ Reaction run in CH_2_Cl_2_; ^6^ Reaction run in PhMe.

**Table 16:** Cycloaddition of azomethine ylide 121 to aldehydes 2 (Scheme 30).

Entry	R^1^	Oxazolines 123 % Yield
1	Ph	89
2	*E*-Ph-CH=CH	84
3	2-pyridyl	90
4	2-furyl	91
5	*i*-Pr	80
6	Et	95
7 ^1^	CO_2_Et	72

^1^ 5 equiv of dipolarophile used.

**Table 17:** Cycloaddition of azomethine ylide **126** to aromatic aldehydes **2** (Scheme 31).

Entry	Ar	Oxazolidines 127 % Yield
1	4-Cl-C_6_H_4_	72
2	Ph	72
3	4-Me-C_6_H_4_	74
4	4-OMe-C_6_H_4_	75
5	4-SMe-C_6_H_4_	70
6	3,4-OCH_2_O-C_6_H_3_	68
7	4-NMe_2_-C_6_H_4_	61
8	4-F-C_6_H_4_	70
9	4-Br-C_6_H_4_	65
10	2-Cl-C_6_H_4_	60
11	3-NO_2_-C_6_H_4_	78
12	4-NO_2_-C_6_H_4_	77
13	2-thienyl	62

**Table 18:** Cycloaddition of azomethine ylide **129** to formaldehyde (**2m**), and subsequent FVT-induced cycloreversion-intramolecular cycloaddition (Scheme 32).

Entry	R^1^	R^2^	Oxazolidines 130 % Yield	Pyrrolidines 131 % Yield
1	Me	H	88	72
2	(CH_2_)_2_CO_2_Me	H	82	82
3	-(CH_2_)_3_-		60	98

**Table 19:** Cycloaddition of azomethine ylide **136** to aldehydes **2** (Scheme 34).

Entry	R^1^	R^2^	Oxazolidines 137a:137b:138c ^1^
1	Me	Ph	86:12:2 ^2^
2	Me	4-OMe-C_6_H_4_	65:35:0
3	Me	4-NO_2_-C_6_H_4_	65 ^3^
4	*t*-Bu	Ph	50:50:0

^1^ Determined by ^1^H-NMR; ^2^ Ratio changed to 16:78:6 on standing in CHCl_3_ for 4 days at rt; ^3^A mixture of three oxapyrrolizidines was obtained from which the single stereoisomer **137a** was isolated in 65% yield.

**Table 20:** Cycloaddition of azomethine ylide **140** to aldehydes **2** (Scheme 35).

Entry	R^1^	Oxazolidines 141 % Yield
1	H	quant.
2	Ph	69
3	4-F-C_6_H_4_	61
4	4-NO_2_-C_6_H_4_	45
5	4-OMe-C_6_H_4_	50
6	2-furyl	51
7	*n*-Pr	86
8	*n*-Bu	80
9	*c*-C_6_H_11_	80

**Table 21:** Cycloaddition of azomethine ylide **160** to aldehydes and a ketone (Scheme 39).

Entry	R^1^	R^2^	Product (% Yield)
1	Ph	H	**163a** (59) + **162a** (11)
2	4-NO_2_-C_6_H_4_	H	**163b** (51) + **162b** (7)
3	2-naphthyl	H	**163c** (28) + **162c** (3)
4	4-OMe-C_6_H_4_	H	**163d** (52) + **162d** (7)
5	Ph	Ph	**163e** (47)
6	*E*-Ph-CH=CH	H	**163f** (59) + **162f** (11)
7	2-pyridyl	H	**163g** (63)

**Table 22:** Cycloaddition of azomethine ylides **167** to aldehyde 2**a** and ketone **3c** (Scheme 40).

Entry	165	Carbonyl Compound	Product 168	Yield, ds Ratio
1	**a** (R^1^ = H, M = SnBu_3_)	PhCHO **2a**	**a**	63%, 1.4:1
2	**b** (R^1^ = *i*-Pr, M = SnBu_3_)	PhCHO **2a**	**b**	56%, 4:1.9:1 ^1,2^
3	**a** (R^1^ = H, M = SnBu_3_)	Ph_2_CO **3c**	**c**	65%
4	**c** (R^1^ = H, M = SiMe_3_)	Ph_2_CO **3c**	**c**	81%

^1^ Diastereoisomers not separated; ^2^ Stereochemistry not determined.

**Table 23:** Cycloaddition of azomethine ylide **172**, formed from silylamine reagent **178**, with aromatic aldehydes **2** (Scheme 45).

Entry	Ar	Product	% Yield
1	Ph	**173a**	95
2	2-NO_2_-C_6_H_4_	**173b**	75
3	4-F-C_6_H_4_	**173c**	89
4	2,4-(F)_2_-C_6_H_3_	**173d**	80
5	2-Br-C_6_H_4_	**173e**	76
6	4-Br-C_6_H_4_	**173f**	86
7	4-CN-C_6_H_4_	**173g**	82
8	4-NMe_2_-C_6_H_4_	**173h**	78
9	4-OMe-C_6_H_4_	**173i**	93
10	3,4,5-(OMe)_3_-C_6_H_2_	**173j**	~100
11	4-NHAc-C_6_H_4_	**173k**	78
12	2,4,6-(Me)_3_-C_6_H_2_	**173l**	80
13	4-CO_2_H-CC_6_H_4_	**173m**	59
14	2-OH-C_6_H_4_	**173n**	54
15	3-OH-C_6_H_4_	**173o**	‒
16	4-OH-C_6_H_4_	**173p**	‒

**Table 24:** Cycloaddition of azomethine ylide **172**, formed from silylamine reagent **178**, with heteroaromatic aldehydes **2** (Scheme 45).

Entry	Aldehyde	Product Oxazolidine	% Yield
1	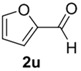	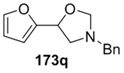	100
2	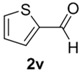	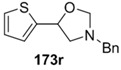	92
3	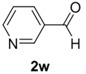	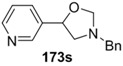	73
4	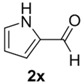	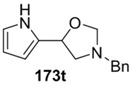	‒
5	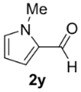	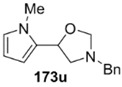	‒
6	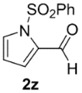	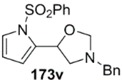	90

**Table 25:**
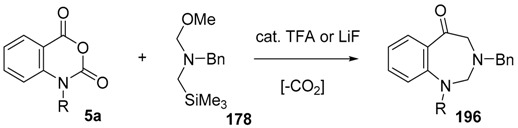
Transformation of isatoic anhydride and *N*-functionalised isatoic anhydrides **5a** into 1,3-benzodiazepin-5-ones **196**.

Entry	R	Method ^1^	Time (h)	% Yield ^2^ 196
1	H	A	24	42
2	Me	A	36	92
3	Et	A	16	79
4	Allyl	A	16	71
5	Bn	A	40	77
6	Ph	A	16	80
7	H	B	3	0
8	Me	B	6	88
9	Et	B	2	96
10	Allyl	B	4	76
11	Bn	B	3	100
12	Ph	B	12	90

^1^ Reaction conditions, Method A: **178** (1.8 equiv.), 4 Å molecular sieves, CF_3_CO_2_H (0.05 equiv.), CH_2_Cl_2_, 0 °C to rt; Method B: **178** (1.8 equiv.), 4 Å molecular sieves, LiF (1.25 equiv.), CH_3_CN, sonication, 35 °C; ^2^ Yield of product isolated after chromatography and/or crystallization.

**Table 26:**
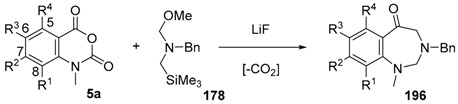
Transformation of benzo-substituted *N*-methyl isatoic anhydrides **5a** into 1,3-benzo-diazepin-5-ones **196** ^1^.

Entry	R^1^	R^2^	R^3^	R^4^	Time (h)	% Yield ^2^ 196
1	H	H	H	H	6	88
2	OMe	H	H	H	3	80
3	H	F	H	H	24	66
4	H	CO_2_Me	H	H	1.5	94
5	H	OMe	H	H	48	- ^3^
6	H	H	Me	H	24	66 ^4^
7	H	H	Cl	H	3	76
8	H	H	Br	H	2	63
9	H	H	OMe	H	41	46 ^5^
10	H	F	F	H	6	93
11	H	H	H	Me	24	- ^6^
12	H	OMe	OMe	H	56	- ^7^

^1^ *Reaction conditions*: **178** (1.8 equiv.), 4 Å molecular sieves, LiF (1.25 equiv.), CH_3_CN, 35 °C, sonication; ^2^ Yield of product isolated after chromatography and/or crystallization; ^3^ 73% of starting material was recovered; ^4^ 13% of starting material was recovered; ^5^ 36% of starting material was recovered; ^6^ 79% of starting material was recovered; ^7^ 77% of starting material was recovered.

**Table 27:** 1,3-Dipolar cycloaddition reaction of phthalic anhydrides **5b** with azomethine ylide **172**, generated in situ from precursor **178** ^1^.

Entry	Phthalic Anhydride 5b	Product 200	% Yield (Isomer Ratio) ^2^
1	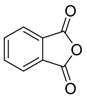	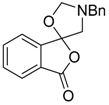	90
2	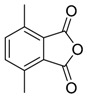	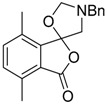	~100
3	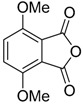	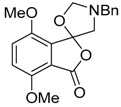	90
4	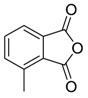	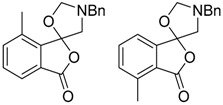	~100 (48:52)
5	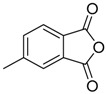	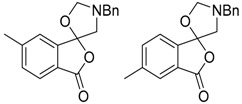	88 (63:37)
6	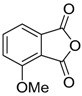	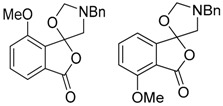	78 (30:70)
7	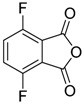	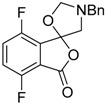	~100
8	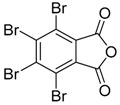	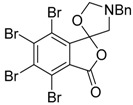	67

^1^ *Reaction conditions*: **5b** (1.0 equiv.), **178** (1.1 equiv.), trifluoroacetic acid (0.05 equiv.), CH_2_Cl_2_ (7.5 mL/mmol **201**), 4Å molecular sieves, N_2_, 0 to 25 °C, 18 h. Column chromatography on Florisil™, eluting with EtOAc; ^2^ Determined by ^1^H-NMR analysis.

**Table 28:** Reductive ring-opening of oxazolidines **200** with NaBH_4_^1^.

Entry	Oxazolidine 200	Isobenzofuranone 201	% Yield ^1^
1	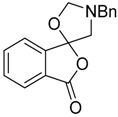	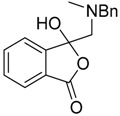	68
2	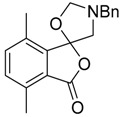	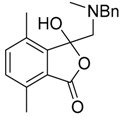	69
3	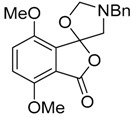	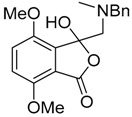	35
4	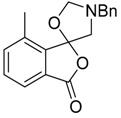	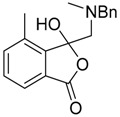	31
	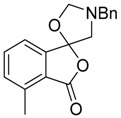	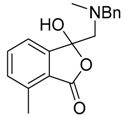	31
5	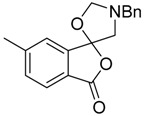	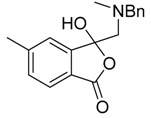	29
	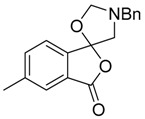	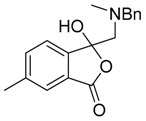	14
6	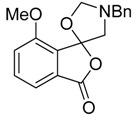	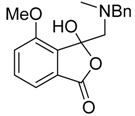	20
	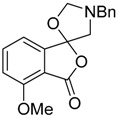	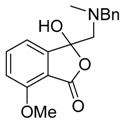	26
7	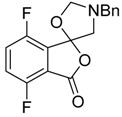	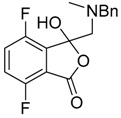	Trace
8	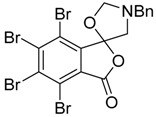	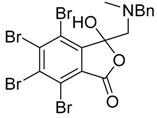	12

^1^ *Reaction conditions*: oxazolidine **200** (1.0 equiv.), NaBH_4_ (1.50 equiv.), MeOH (6.0 mL/mmol **200**), 25 °C, N_2_, 4–18 h.

**Table 29:** Reaction of imines **217**, ethyl diazoacetate (**218**) and Cu(tfacac)_2_ in CH_2_Cl_2_ at 40 °C (Scheme 61).

Entry	R^1^	R^2^	% Yield 219 Endo/Exo
1	4-OMe-C_6_H_4_	4-Br-C_6_H_4_	41/0
2	Ph	4-Br-C_6_H_4_	39/0
3	4-OMe-C_6_H_4_	4-Cl-C_6_H_4_	27/0
4	4-Me-C_6_H_4_	4-Br-C_6_H_4_	40/0
5	*E*-Ph-CH=CH	4-Br-C_6_H_4_	18/0
6	4-CN-C_6_H_4_	4-Br-C_6_H_4_	17/20
7	4-NO_2_-C_6_H_4_	4-Br-C_6_H_4_	29/9
8	3-NO_2_-C_6_H_4_	4-Br-C_6_H_4_	11/9
9	*E*-Ph-CH=CPh	Ph	0/0
10	4-OMe-C_6_H_4_	4-OMe-C_6_H_4_	0/0
11	4-OMe-C_6_H_4_	Ph	0/0
12	Ph	Ph	0/0
13	4-NO_2_-C_6_H_4_	4-OMe-C_6_H_4_	0/0

**Table 30:** Reaction of benzaldehyde-derived imines **210** with difluorocarbene and aldehydes **2** or ketones **3**, yielding oxazolidinones **222** (Scheme 63).

Entry	R^1^	R^2^	R^3^	Product	% Yield (*dr*)
1	Ph	Ph	H	**222a**	39 (25:14)
2	CO_2_Et	Ph	H	**222b**	25 (3:2)
3	SiMe_3_	Ph	H	**222c**	37 (2:1)
4	SiMe_3_	2-(CHO)-C_6_H_4_	H	**222d**	13 (10:3)
5	Ph	CH_3_	H	**222e**	43 (1:1)
6	Ph	CH_3_	CH_3_	**222f**	3
7	Ph	Ph	CH_3_	**222g**	-

**Table 31:** The reaction of imines **223c**, difluorocarbene and trifluoromethylketones **3** (Scheme 67).

Entry	Ar^1^	Ar^2^	Ar^3^	% Yield 238
1	Ph	Ph	Ph	66
2	4-Cl-C_6_H_4_	4-Cl-C_6_H_4_	Ph	61
3	4-Cl-C_6_H_4_	4-CN-C_6_H_4_	Ph	10 ^1^
4	4-CF_3_-C_6_H_4_	4-CF_3_-C_6_H_4_	Ph	12
5	3-CF_3_-C_6_H_4_	3-CF_3_-C_6_H_4_	Ph	38
6	Ph	Ph	4-CH_3_-C_6_H_4_	63
7	Ph	Ph	4-Cl-C_6_H_4_	54
8	Ph	Ph	4-F-C_6_H_4_	76
9	Ph	Ph	3-CF_3_-C_6_H_4_	74

^1^ Mixture of stereoisomers.

**Table 32:** Intramolecular cycloaddition of ylide and carbonyl esters within **5d** yielding epoxybenzodiazepines **240** (Scheme 68) ^1^.

Entry	Imine 217	R^1^	R^2^	Product	% Yield
1	**a**	Ph	Ph	**240a**	74
2	**b**	Ph	4-Ome-C_6_H_4_	**240b**	92
3	**c**	Ph	4-CN-C_6_H_4_	**240c**	70
4	**d**	Ph	2,4-(Cl)_2_-C_6_H_3_	**240d**	73
5	**e**	Ph	1-naphthyl	**240e**	75
6	**f**	Ph	CH_2_=Cme	**240f**	77
7	**g**	Ph	(*E*)-Me-CH=CH	**240g**	68
8	**h**	Ph	(*E*)-Ph-CH=CH	**240h**	56
9	**i**	Ph	(*E*)-Ph-CH=CPh	**240i**	70
10	**j**	4-Br-C_6_H_4_	2-Furyl	**240j**	75
11	**k**	4-Br-C_6_H_4_	Ph	**240k**	85
12	**l**	2,4-(Cl)_2_-C_6_H_3_	Ph	**240l**	70
13	**m**	(4-Cl-C_6_H_4_)_2_-CH	Ph	**240m**	88
14	**n**	Me_3_SiCH_2_	Ph	**241n**	67

^1^ *Reagents and Conditions*: Pb (2 equiv.), Bu_4_NBr (2 equiv.), imine **217** (1 equiv.), CBr_2_F_2_ (3 equiv.), ultrasound, 45 °C.

**Table 33:** Intramolecular cycloaddition of dichlorocarbene derived ylides and ester carbonyl **5e** yielding epoxybenzodiazepinones **241** (Scheme 71) ^1^.

Entry	Imine 217	R^1^	R^2^	Method ^1^	Product 241	% Yield
1	**b**	Ph	4-OMe-C_6_H_4_	A	**b**	82
2	**b**	Ph	4-OMe-C_6_H_4_	B	**b**	92
3	**o**	4-Br-C_6_H_4_	4-OMe-C_6_H_4_	A	**o**	97
4	**d**	Ph	2,4-(Cl)_2_-C_6_H_3_	A	**d**	56
5	**p**	4-Br-C_6_H_4_	4-CN-C_6_H_4_	B	**p**	74
6	**q**	Ph	2-furyl	B	**q**	47
7	**i**	Ph	(*E*)-Ph-CH=CPh	A	**i**	100
8	**k**	4-Br-C_6_H_4_	Ph	B	**k**	60

^1^ Method A: **217**, CHCl_3,_ KOH, BnEt_3_NCl, 20 °C. Method B: **217**, Cl_3_CCO_2_Na, BnEt_3_NCl, CHCl_3_, 60 °C.

**Table 34:** Reduction of cycloadducts **241** and **240** with LiAlH_4_ (Scheme 72).

Entry	Starting Material	R^1^	R^2^	Method ^1^	Product 247	% Yield
1	**241b**	Ph	4-OMe-C_6_H_4_	A	**b**	77
2	**241o**	4-Br-C_6_H_4_	4-OMe-C_6_H_4_	A	**o**	47 ^2^
3	**241i**	Ph	(*E*)-Ph-CH=CPh	A	**i**	68
4	**240a**	Ph	Ph	B	**a**	65
5	**240m**	(4-Cl-C_6_H_4_)_2_CH	Ph	B	**m**	70

^1^ Method A: LiAlH_4_, Et_2_O, 34 °C. Method B: LiAlH_4_, 1,4-dioxane, 101 °C; ^2^ The conversion of lactam **241o** was 75%.

**Table 35:** Multicomponent reaction of aldehydes **2**, isocyanides **248** and trialkylboron reagents **249** (Scheme 73).

Entry	R^1^	R^2^	R^3^	Cycloadduct 251 % Yield
1	4-OMe-C_6_H_4_	4-Cl-C_6_H_4_	Et	85
2	4-Me-C_6_H_4_	4-Cl-C_6_H_4_	Et	67
3	Ph	4-Cl-C_6_H_4_	Et	22
4	4-Cl-C_6_H_4_	4-Cl-C_6_H_4_	Et	65
5	4-F-C_6_H_4_	4-Cl-C_6_H_4_	Et	36
6	cyclohexyl	4-Cl-C_6_H_4_	Et	-
7	1-cyclohexenyl	4-Cl-C_6_H_4_	Et	-
8	PhCH_2_	4-Cl-C_6_H_4_	Et	-
9	TsCH_2_	4-Cl-C_6_H_4_	Et	-
10	4-OMe-C_6_H_4_	4-OMe-C_6_H_4_	Et	50
11	4-OMe-C_6_H_4_	3-pyridyl	Et	62
12	4-OMe-C_6_H_4_	Me_3_C	Et	-
13	4-OMe-C_6_H_4_	EtO_2_C	Et	7
14	4-Cl-C_6_H_4_	4-Cl-C_6_H_4_	Bu	40
